# When crops fail, forests follow: Agricultural shocks and deforestation in Zambia

**DOI:** 10.1073/pnas.2427156122

**Published:** 2025-10-03

**Authors:** Pablo J. Ordóñez, Protensia Hadunka, Gemma Del Rossi, Kathy Baylis

**Affiliations:** ^a^Inter-American Development Bank, Office of Strategic Planning and Development Effectiveness (SPD), Washington, DC 20577; ^b^Department of Agricultural and Consumer Economics, University of Illinois at Urbana-Champaign, Urbana, IL 61801; ^c^Bren School of Environmental Science and Management, University of California, Santa Barbara, CA 93117; ^d^Department of Geography, University of California, Santa Barbara, CA 93117

**Keywords:** deforestation, agricultural productivity, fall armyworm, charcoal, Zambia

## Abstract

As climate change makes agricultural production shocks more frequent and severe, it is vital to understand their effect on farmer welfare, land use, and deforestation. Theoretically, a change in agricultural productivity could increase or decrease deforestation by changing demand for agricultural land and/or through the consumption of forests as a coping strategy. This paper uses the introduction of a crop pest to sub-Saharan Africa to estimate the effect of a negative agricultural productivity shock on deforestation. Using primary household data, we first find that farmers who were exposed to higher levels of fall armyworm saw substantial decreases in yield and food security. Using estimates of fall armyworm suitability in conjunction with machine-learning models of maize yields and deforestation, we find that the introduction of the fall armyworm induced a doubling of the deforestation rate in Zambia in the 3 y following the outbreak. This increase was driven both by increased agricultural land expansion and increased charcoal production as a coping strategy. These responses vary substantially over space. More remote areas experienced 23% lower FAW-induced deforestation compared with the sample average, suggesting that farmers with access to maize and charcoal markets may have increased deforestation as a response. Wealthier areas were also less likely to deforest in response to FAW pressure. In sum, our results suggest that negative agricultural productivity shocks may lead to a negative climate feedback, with farmers engaging in emissions-increasing strategies in response.

## Significance

This article estimates how farmers respond to a negative agricultural production shock from an invasive pest. While one might think that decreasing the productivity of agricultural land would lead to lower demand for agricultural land and thus lower rates of land conversion, we find that those farmers facing the largest pest outbreak deforest more through expanding agricultural land and increasing charcoal production. This effect was particularly pronounced for farmers who have greater access to markets, but lower for wealthier households. Along with related work, our results suggest that households with more available mitigation options are less likely to turn to charcoal as a coping strategy.

Climate change is expected to negatively impact agricultural productivity, with particularly severe consequences for agricultural producers in the global tropics (1–3). Higher temperatures, increasing frequency and severity of floods and droughts along with expanded insect threats all have the potential to reduce agricultural yields. These drops in productivity will likely affect land use, which will in turn affect carbon emissions. But how agricultural productivity shocks affect land use is unknown. A decrease in agricultural productivity may 1) reduce land in agriculture in response to the decrease in productivity (4–6) or 2) expand agricultural land to make up for decreased production per acre (7, 8) and/or 3) lead farmers to consume forests as an alternate source of income (9). In this paper, we explore how a shock to agricultural productivity affects deforestation. We use the introduction of *Spodoptera frugiperda* J.E Smith (Fall Armyworm or FAW), to sub-Saharan Africa (SSA), to test how farmers respond to a negative agricultural shock in terms of changes in cropped land and forest consumption.

**Fig. 1.**
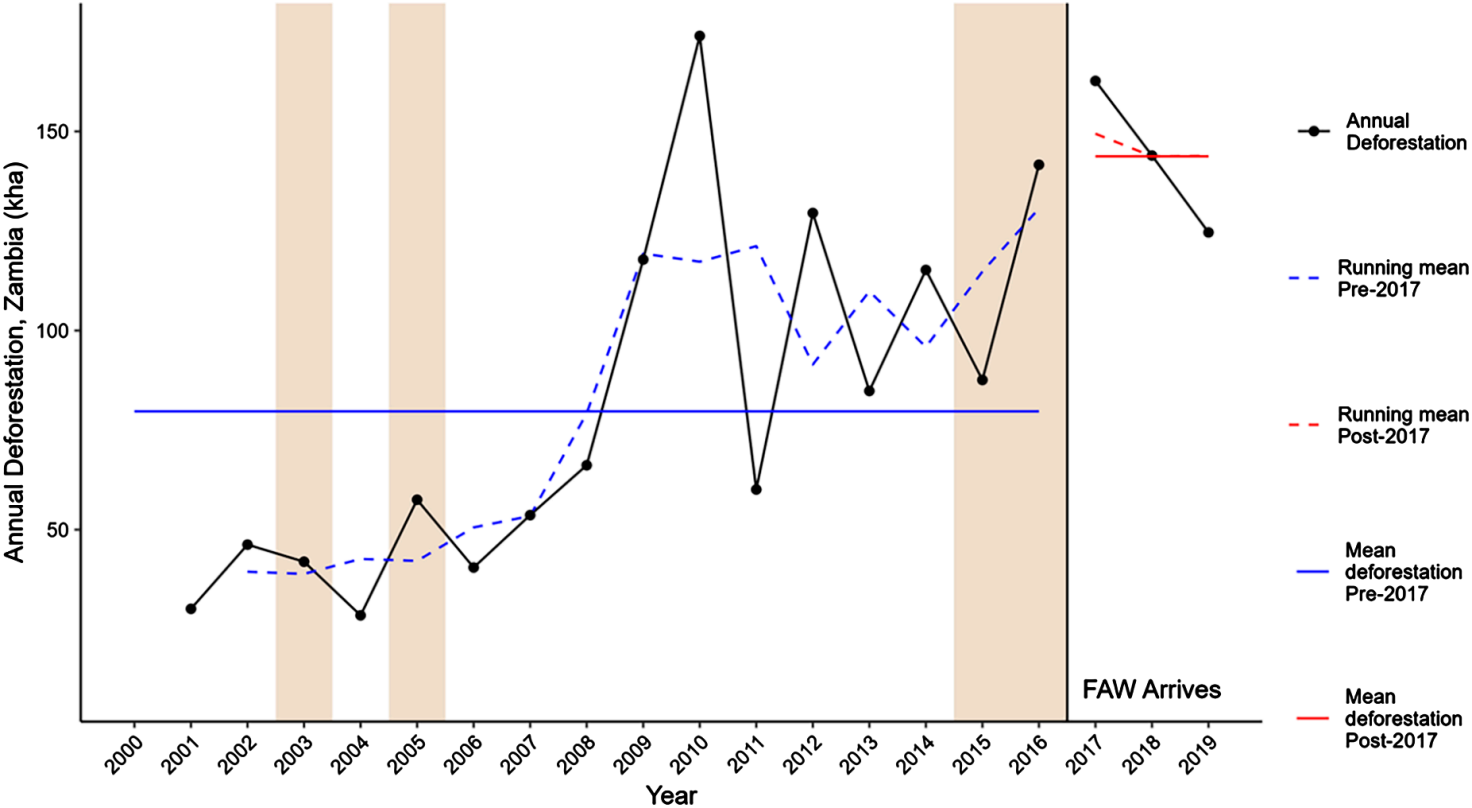
Total estimated deforestation in Zambia from Global Forest Watch, 2001 – 2019. Notes: Source (17). The shading indicates drought conditions (Standardized Precipitation Index (SPI) and Standardized Precipitation-Evapotranspiration Index (SPEI) < 0 on average across Zambia) for that year using Climate Hazards Center InfraRed Precipitation with Station data (CHIRPS) (18). 3-y running means of annual deforestation (kha) are included to smooth RS inconsistencies and delays in detection from disturbances such as late-year fires.

Forests are important for protecting biodiversity, supporting livelihoods as well as combating climate change. Deforestation is a primary driver of net anthropogenic carbon dioxide (CO_2_) emissions, with estimated global gross emissions of 8.1 ± 2.5 GtCO_2_e yr−1 (mean ± s.d.) greenhouse gas (GHG) emissions from deforestation and other disturbances (10). The IPCC special report on Climate Change and Land estimates that global deforestation contributes to 13% of total GHG, while agriculture, forestry, and other land uses contribute an additional 23% to overall anthropogenic GHG emissions (11). In SSA, cropland expansion by smallholders is a main cause of deforestation (12–15) along with fuelwood harvesting, particularly for charcoal (16). In Zambia, yearly deforestation between 2001 and 2019 was on average 89.1 thousand ha per year, for a total loss of 1.58 million hectares during this period (Fig. 1). Between 2001 and 2016, average deforestation was 79.8 thousand ha per year (about 0.3% per year), but starting in 2016 there was a significant break from this trend, and the average between 2017 and 2019 is approximately twice as large as that of the 2001–2016 period.

FAWs are an insect pest, native to South America that are particularly damaging to maize, the primary staple crop throughout most of SSA (19). FAW were first observed in West Africa in 2016, with early reports coming from Nigeria, Togo, Benin, and the island of São Tomé (20). They were observed in Zambia by November of the same year (21) and by 2018, 44 countries in SSA had reported infestations (19). FAW attack young maize plants, and dig into the cobs of older plants, decreasing maize crop yields from 35% to over 50% if not controlled (19, 20). The cost of FAW to Zambian maize production is substantial, with an estimated 1.2 million tons lost in 2017, amounting to 39% of the total production for Zambia that year (22). Although FAW prefer maize, they also attack other crops, such as sorghum, soybeans, and rice (23). The continued need for active management of FAW through pesticide applications (23) is expected to increase production costs, implying this is not just a transitory shock to maize cultivation, but a long-run downward shift in agricultural productivity and, like other insect pests, is predicted to become more of a problem with climate change (24, 25). We want to understand the ways in which these climate-driven pest infestations affect deforestation which in turn could increase greenhouse gas emissions, furthering climate change.

The effect of a shock to agricultural productivity on deforestation is not clear ex-ante. Increased agricultural productivity may lead to agricultural land expansion due to the increased profitability of land in agriculture. On the other hand, by allowing more production per acre, increased agricultural productivity can reduce the pressure for agricultural land (Borlaug’s hypothesis). The empirical evidence is mixed (26). Past literature has found that increases in agricultural technology have decreased deforestation (27–29) while Angelsen (30) and others demonstrate that increased agricultural productivity driven by commodity prices can increase deforestation (31–33). Other works have used negative agricultural production shocks to explore their effect on consumption of forests as a coping strategy (34), where in certain cases forest consumption increased and agricultural land expansion did not (9, 35, 36). This paper examines whether a negative shock on agricultural productivity increases or decreases in deforestation. If Borlaug’s hypothesis holds, i.e. that increased productivity drives agricultural land contraction, we might expect to see a negative shock increase agricultural land expansion. On the other hand, farmers might use deforestation as a coping strategy. We test for both potential effects estimating the effect of the introduction of FAW on the change in agricultural land and deforestation driven by charcoal.

We combine primary longitudinal data from farmers before and after the introduction of FAW to understand its effect on yields, food security, and charcoal production. One challenge to identifying the effect of FAW is that because of their rapid spread, all of Zambia was subject to the pest in the 2016/17 agricultural season, so we cannot use FAW roll-out over time and space as a source of identification. Second, FAW presence and maize yields are correlated across space. Thus, we need to carefully consider what would have happened to maize yields and deforestation in the absence of FAW. To address these challenges, we generate a counterfactual by predicting what would have happened to deforestation and yields in Zambia in the absence of the FAW. We train a machine-learning model to predict deforestation and yields, using data before the arrival of the FAW, and including a high-dimensional set of explanatory variables and interactions for weather, terrain, potential yield, accessibility, and population. This allows us to do two things: First, since we are only using data before the arrival of the FAW to train the models, the estimated relationships between deforestation (and yields) and the explanatory variables are not ‘contaminated’ by how these variables might affect deforestation through the effect they might have on the presence of the FAW. Second, since these models are designed to generate accurate out of sample predictions, we can include a large set of potential explanatory variables to capture nonlinearities and threshold effects of deforestation and agricultural production, where these variables will be selected based on their predictive contribution. This approach allows us to have a much more flexible specification than what would be possible in a standard linear regression setting.

As a measure of exposure to the FAW threat, we use a map of FAW suitability from Early et al. (37), and we compare this suitability map against self-reported damage and yields in our primary data to test whether the suitability estimates capture the location of FAW damage. Next, to estimate the impact of FAW, we compare the errors in prediction between our counterfactual machine learning models and the observed yields and deforestation across locations more and less suitable for FAW. Last, we use our results to examine the heterogeneity of FAW impacts across several channels to uncover possible mitigation strategies.

## Results

### Household-Level Impacts of FAW.

**Fig. 2.**
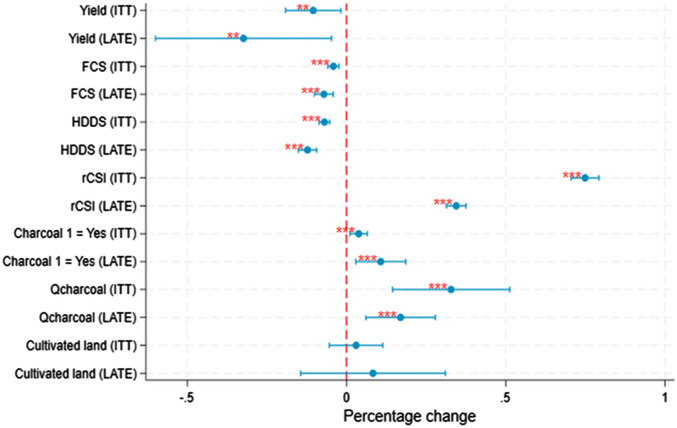
Effect of FAW on yields, charcoal production, and food security from primary data. Notes: Farmer reports of FAW infestations on crop damage are classified into three categories: low (< 25% damage), moderate (25 to 50% damage), and severe (> 50%). Each row corresponds to the estimated effect of a one-category increase in severity of FAW during the prior agricultural growing season, estimated in separate regressions, with 95% CI depicted by the blue lines. Statistical significance is indicated by*** *P* < 0.01, **P < 0.05, *P < 0.1. Regressions are for both the village average FAW infestation level (denoted as ITT) or for the farmer’s infestation level as instrumented by the village average (denoted as LATE). All regressions include controls for growing season rainfall (in mm) and average temperature (in degrees C) and their squares. Household and year fixed effects are included in all models to account for unobserved heterogeneity and temporal variation. For the regression on the probability of charcoal production, we use a correlated random effects (CRE) probit model to incorporate fixed effects. The reported results are in terms of percentage point changes. We estimate food security measures and quantity of charcoal produced using a Tobit model with random effects to account for truncation around zero. For the regressions on the quantity of charcoal produced, maize yields, and food security, we apply the inverse hyperbolic sine transformation, while the regression on cultivated land uses a log transformation. All other regressions are estimated using a linear two-way fixed effects model. In the maize yield regressions, we include input variables as controls. For the LATE estimates, all instruments—except for the regression on the quantity of charcoal produced—are identified as strong predictors of farmers’ self-reported FAW incidence (*SI Appendix*, Table S5). In all the regressions, SE are clustered at the camp level, where camps are a unit used for government agricultural extension and usually contain around five traditional villages. This study randomly selected households from agricultural camps and villages. Detailed first-stage results are presented in the *SI Appendix*, section S1.2. Leads were insignificant across all specifications (see *SI Appendix*, section S1.3 for details).

We first use primary data on a panel of smallholder farmers across Zambia (38) to estimate the effect of FAW on household yield and food security to gauge whether the infestation had an impact on household welfare. We use two measures of being affected by FAW. First, to reduce the potential reporting bias, we estimate the effect of the average number of farmers reporting FAW in the surrounding village, stripping out the farmer’s own reports (denoted as ITT or Intent to Treat in Fig. 2). Second, we use that average level of FAW nearby as an instrument for own-reported FAW (denoted as LATE, or the Local Average Treatment Effect), where these measures represent farmer’s own reports of FAW as predicted by nearby FAW pressure. FAW intensity is measured using farmer self-assessments of crop damage which are categorized as low (<25%), medium (25 to 50%), and high (>50%). Marginal effects of a category increase in FAW intensity are shown in Fig. 2 and full results are presented in *SI Appendix*, Table S1.2. We find that a one-unit increase in FAW severity in the village is associated with a 10% decrease in maize yields, and for self-reported infestations, that loss increases to 32%. This finding is in line with other studies that suggest a 39% loss in yield due to FAWs in Zambia in the first year of their infestation (22). The arrival of FAW is also associated with a notable decrease in household welfare as measured by food security indicators. We find that FAW decreased measures of dietary quality between 7% (LATE - FCS) and 12% (LATE - HDD) and increased the use of coping strategies (rCSI) between 31 and 62%, depending on the estimate used.

We then estimate how farmers respond to this shock. We first examine how having one’s prior harvest affected by FAW affects the probability and quantity of charcoal harvested by a farming household. While FAW can be seen in young maize plants, they are most often observed around February or March, after planting in November. Charcoal production occurs around October, after harvest and before the rainy season and planting begins. Thus, agricultural productivity shocks experienced at harvest may affect households’ decisions to produce charcoal leading up to the next agricultural season. We use a probit with correlated random effects to control for time-invariant household characteristics to estimate changes in probability and a tobit with random effects model to estimate on the quantity of charcoal produced (*SI Appendix*, Table S5). We observe that an increase in FAW intensity increases the probability of producing charcoal between 4 to 11 percentage points (equivalent to a 17 to 49% increase from the baseline probability of 22%). A one-category increase in FAW intensity increases the average quantity of charcoal production by 33% (ITT estimate - for an increase in the village average FAW), or 17% (LATE estimate – for an increase on farm).

Second, we estimate how FAW affects the amount of farmland cultivated by a household in the following agricultural year (*SI Appendix*, Table S5). Our results suggest that households experiencing larger FAW infestations in the prior year expand cultivated land by around 3% per year (ITT estimate), or by 8% per year for self-reported infestations (LATE estimate). However, these estimates are not statistically significant at conventional levels, indicating we cannot rule out the possibility of zero effect. While the direction of these point estimates is consistent with our findings from the grid cell level analysis below, the household-level results for land expansion are not conclusive.

The results from our primary data are suggestive that Zambian smallholder farmers were substantially impacted by the introduction of FAW through reduced yields and increased food insecurity and that at least some of them turned to increasing charcoal production and/or increasing the amount of agricultural land in response. To evaluate how these actions translate to observed deforestation, we introduce a measure of potential FAW suitability (37) to estimate differential levels of household-level FAW vulnerability across space. This measure is a time-invariant index that predicts the suitability of a given area for the year-round presence of the FAW, based on the existing climatic conditions and land use conditions derived from the pest’s native range in the Americas.

We next test whether the FAW suitability estimates capture the FAW damage and yields reported by our household sample (Table 1), to understand whether this suitability index will allow us to capture the negative agricultural productivity shock at the country level, and whether we can use it in our country-wide analyses of the effects of the FAW on deforestation and cropland. As above, the reported damage is a categorical variable between 1 and 3, with the highest value reported for the highest level of damage. We find that households located in areas with a higher FAW suitability report a higher level of incidence and damage in 2018 and 2019. For yield and harvest, we find negative effects for all years between 2017 and 2019, although these results are not significant at conventional levels. Our results suggest that moving from zero to the median FAW suitability of Zambia (index of 80.7) is associated with a loss in yield of between 20 and 40% with respect to 2016. These findings make us confident that the suitability index captures FAW pressure felt by households and allows us to use it in our analysis of the effect of FAW on country-wide deforestation and cropland expansion.

**Table 1.** FAW suitability and crop damages at the household level

	Dependent variable: (SE in parentheses)			
	Reported FAWPresence	ReportedFAW Damage	ln(Yield)(kg/ha)	ln(Total harvest)
2017 × FAW Suitability	0.0001 (0.0017)	−	−0.0060 (0.0041)	−0.0025 (0.0036)
2018 × FAW Suitability	0.0013 (0.0017)	0.0034 (0.0038)	−0.0027 (0.0041)	−0.0028 (0.0036)
2019 × FAW Suitability	0.0002 (0.0019)	0.0084* (0.0043)	−0.0027 (0.0046)	−0.0063 (0.0040)
Observations	2,976	2,232	2,723	2,771

Notes: FAW suitability is measured using a scale of 1 to 100 from ref. 37. The marginal effects therefore represent the effect of a 1-unit increase in FAW suitability. Statistical significance is indicated by ***P < 0.01, **P < 0.05, and *P < 0.1. The SE are clustered at the camp level.

### Machine Learning Approach.

**Fig. 3.**
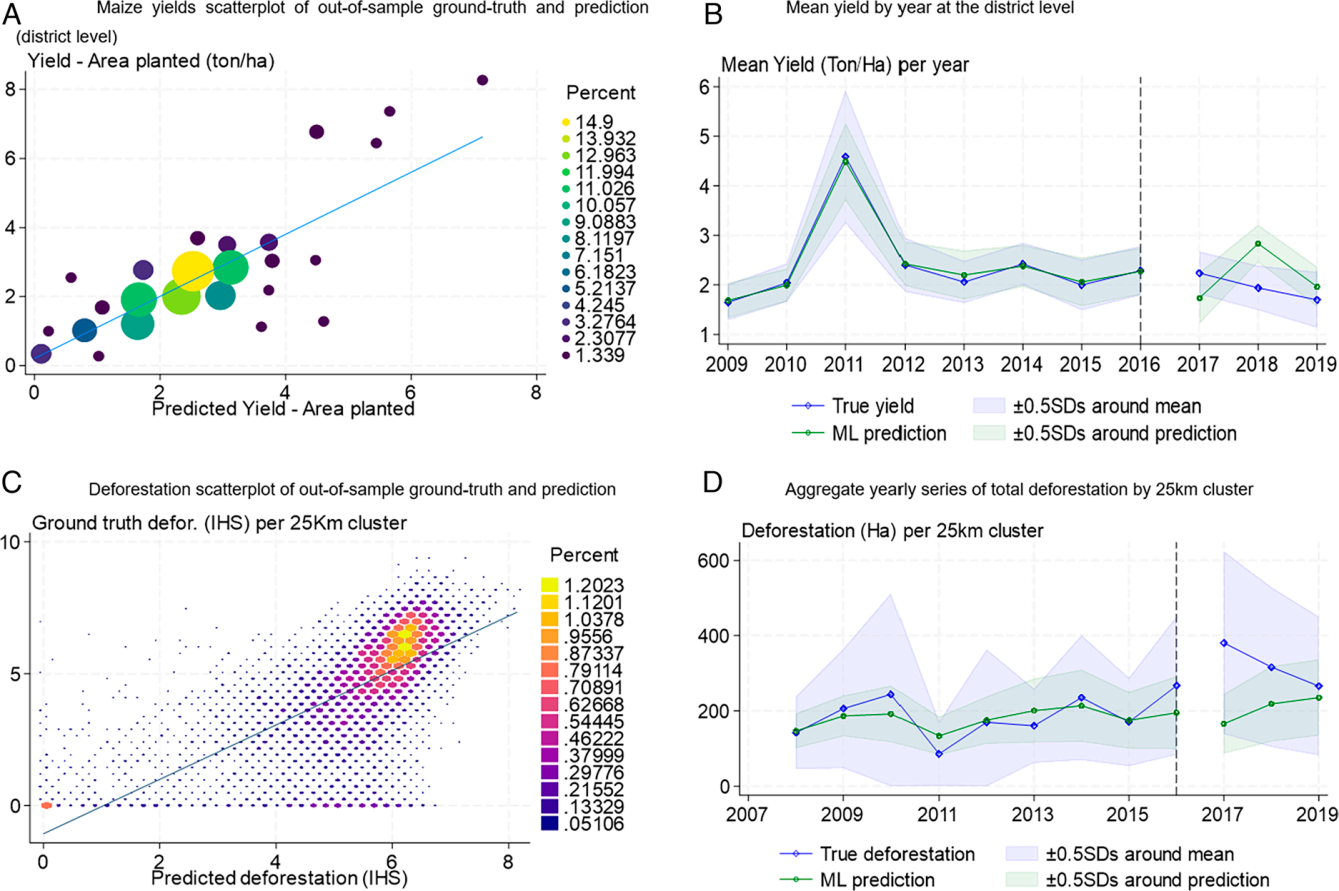
ML model predictions for yield and deforestation. Notes: This figure shows the goodness of fit of the two ML models we trained, with (*A* and *B*) for the model trained to predict the maize yields at the district level, and (*C* and *D*) for the model trained to predict deforestation at the grid cell level, both on the validation sample (95% of the sample). Panel *A* shows the scatterplot of the observed yield (*y*-axis) and the predicted yield (*x*-axis) on the validation sample (20% of the sample), and the size and brightness of the circles represent the areas with a higher density of points. Panel *B* shows the mean prediction error per year (observed yields minus predicted yields), conditional on monthly rainfall and temperature in the current and previous year. Panel *C* shows the scatterplot of the observed yield (*y*-axis) and the predicted yield (*x*-axis) for the whole sample, and the size and brightness of the hexagons represent the areas with a higher density of points. Panel *D* shows the time series of the average deforestation per year, with data aggregated from the grid cell level into 25 × 25 km clusters of grid cells, both for the predicted and the observed deforestation.

We next train two machine learning (ML) models, one for predicting maize yields at the district level using data from the Zambian Central Statistics Organization (39), and another to predict deforestation at the grid cell level from Hansen et al. (17). Both models perform well in out of sample prediction, with an R-squared of 0.91 for yield and 0.89 for deforestation when evaluated at a more aggregate level (25 × 25 km cluster of grid cells) and 0.42 at the grid cell level (Fig. 3). The maize yield ML model slightly underpredicts the true yield (Fig. 3*A*), especially for the higher end of the distribution of observed maize yields, but it performs very well for the middle of the distribution. In aggregate, we observe that the average yearly difference between the observed and predicted maize yields is not statistically different from zero for the pre-FAW period (Fig. 3*B*).

We use predicted maize yields in our ML model for deforestation to control for the effect of weather on deforestation through its effect on maize yields. Our ML model for deforestation also performs well in the validation sample (Fig. 3*A*). We can see that for the average grid cell, the predicted forest loss by grid cell and year follows a similar trend to the ground truth forest loss prior to the introduction of FAW (Fig. 3*D*).

**Fig. 4.**
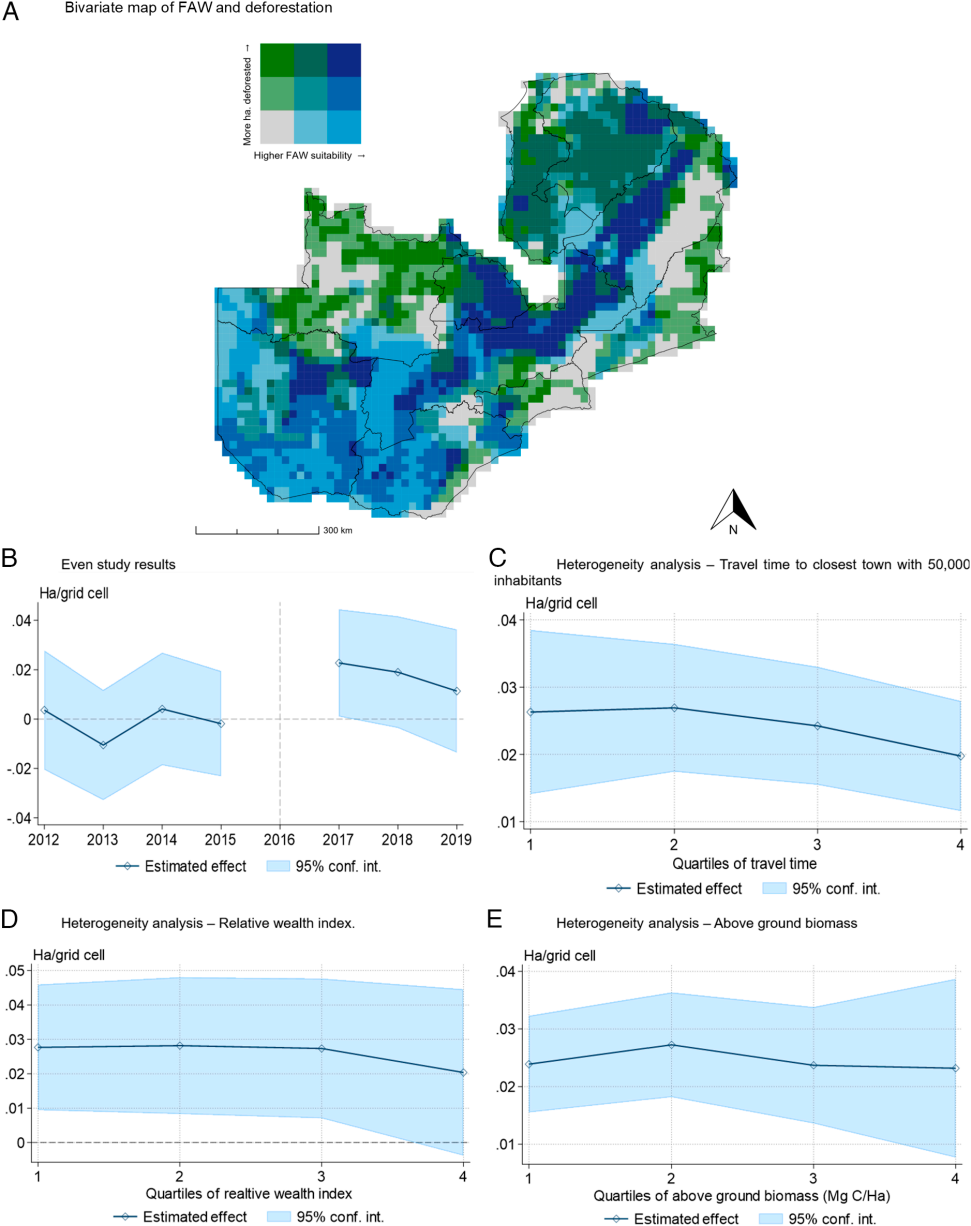
ML event study on deforestation and heterogeneity. Notes: Panel (*A*) is a bivariate choropleth map of the FAW suitability index (*x*-axis) and total deforestation between 2017 and 2019 (*y*-axis). The model for Panel (*B*) has the deforestation prediction error as the outcome variable, and includes the predicted maize yield, as well as year and grid cell fixed effects. The SE are estimated through the spatial bootstrapping procedure explained in the Methodology section in the *SI Appendix*, section S3.3. Panels (*C*–*E*) are estimated in a regression where the outcome variables is also the deforestation prediction error, and the estimated coefficients come from the interaction of a dummy variable that takes the value of 1 for every after 2016 (Post = 1 if year >2016), the FAW suitability index, and a factor variable with four values (1 to 4) that denote the quartile each grid cell for the variable of interest. The variables in the heterogeneity analysis panels are derived using data from refs. 38, 40 and 41, respectively.

For deforestation, we find that after the arrival of the FAW in 2016, there is an increase in deforestation in grid cells that are more suitable for the year-round presence of the FAW, as expressed by the suitability index. Evaluated at the median suitability level (80.7), the arrival of the FAW led to an increase in deforestation of 1.53 ha per grid cell (mean grid cell area is ~480.6 ha) (Fig. 4*B* and *SI Appendix*, Table S10), which translates to a 102% increase in the deforestation rate. Importantly, we find no evidence of different pretrends in deforestation by FAW suitability, since all the estimated coefficients pre-2017 are not significantly different from zero. Additionally, the estimated effect when we use the prediction error in deforestation as our outcome variable is smaller than what we find when using observed deforestation (*SI Appendix*, Table S10). This finding is an indication that, as we explain in our method section, there are factors that affect deforestation that are likely correlated with FAW suitability that we do not properly capture in our regression model, leading to bias.

FAW-driven deforestation differs by location. First, we find that in more remote areas (e.g. approximately 20 h of walking time to the nearest town with 50,000 inhabitants), the increase in deforestation from FAW was approximately 14% lower than what it was for the whole sample of grid cells, although it is still significantly different from zero (Fig. 4*C*). This result may reflect the lack of access to charcoal markets in more remote locations. These more remote areas are also less populated and poorer and have a lower potential for maize yield (*SI Appendix*, Fig. S3). Second, the wealthiest areas see a smaller (33% lower) loss in forest cover in response to FAW (Fig. 4*D*). We see in the areas with the highest levels of above ground biomass, the arrival of the FAW led to a smaller increase in deforestation. These are areas that on average are more remote, have higher tree cover, and lower potential maize yield (*SI Appendix*, Fig. S3). Finally, we also find that most of this heterogeneity is driven by areas where the potential maize yield is lower, which are areas that are less suitable for other crops and where the effects of the FAW on cropland expansion are smaller (*SI Appendix*, Fig. S4).

**Fig. 5.**
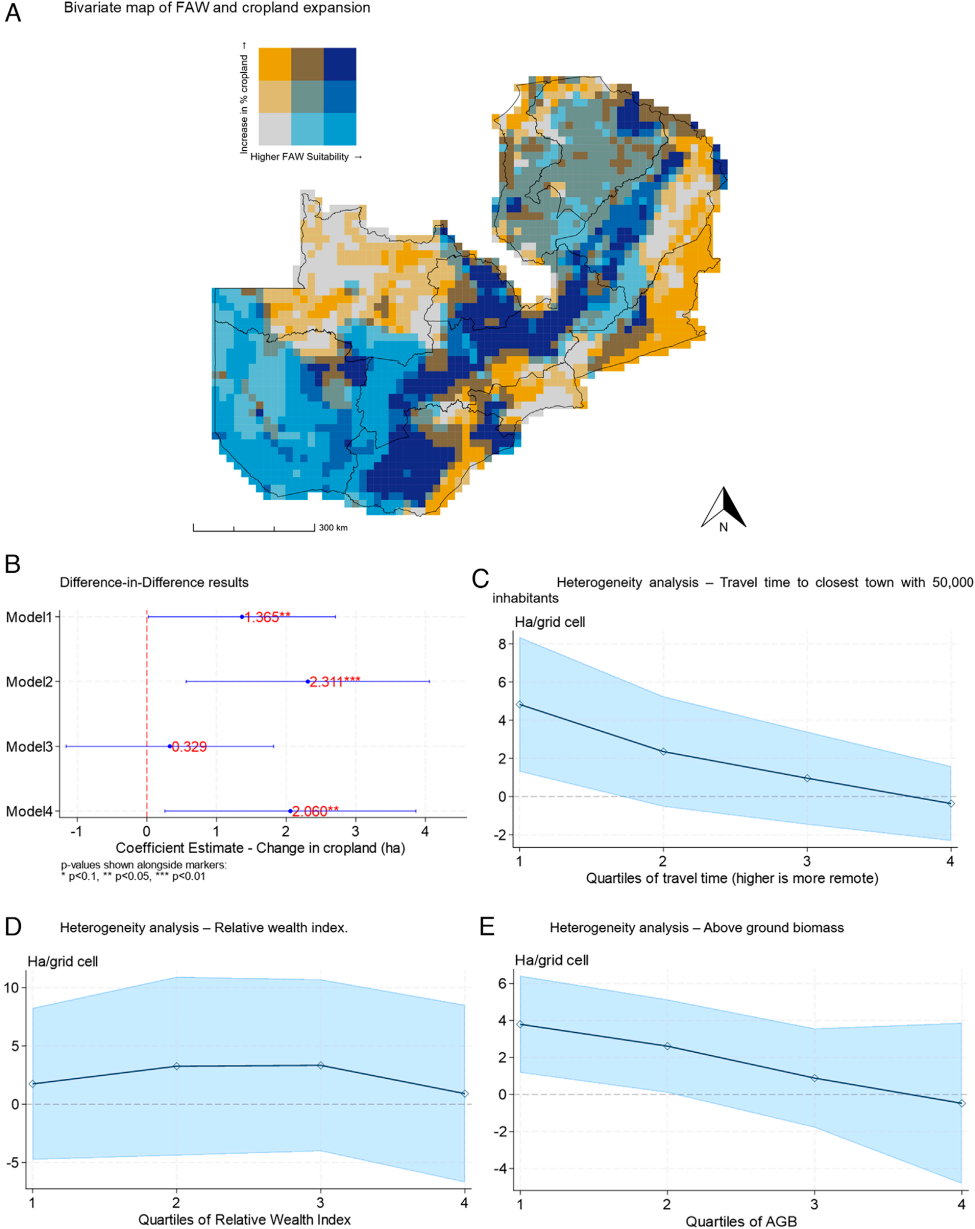
Cropland expansion and heterogeneity. Notes: Panel (*A*) is a bivariate choropleth map of the FAW suitability index (*x*-axis) and the percentage increase in cropland expansion between 2015 and 2019 (*y*-axis). The models from Panel (*B*) are regression models where the outcome variable is the cropland area (in hectares) per grid cell, and for all the models, we include year and grid cell fixed effects, with SE clustered at the district level. Model 1 includes no covariates. Model 2 includes mean rain and temperature by grid cell, both contemporaneous and lagged 1 y. Model 3 includes the predicted maize yield per grid cell and year. Model 4 includes the predicted maize yield, as well as the mean rain and temperature (both contemporaneous and lagged 1 y). Our preferred specification is Model 4, and this is the one that we use for the heterogeneity analysis (Panels *C*–*E*), where the regressions in each case include year and grid cell fixed effects. The variables in the heterogeneity analysis panels are derived using data from refs. 38, 40 and 41, respectively.

As a complement to our ML deforestation analysis, we examine how the arrival of the FAW affected cropland expansion using data from Potapov et al. (42). We find that grid cells where the FAW suitability index is above the median (80.7), see an increase in cropland post-2016 of between 0.3 and 2.3 ha per grid cell (Fig. 5*A*). Under our preferred specification (Model 4), the estimated effect of moving from 0 to the index median is 2.06 ha per grid cell, or a 9.8% increase in cropped area relative to the 2015 baseline. This effect is largest in areas that are closer to town and in locations with less baseline above-ground biomass (Fig. 5 *B* and *D*), both of which are correlated with locations that have fewer trees and higher potential yield (*SI Appendix*, Fig. S3). Most of the effect of FAW on cropland expansion is driven by grid cells in the middle of the wealth distribution—thus neither households that are very poor nor very wealthy, as measured by the relative wealth index (Fig. 5*C*), and by grid cells where the potential maize yield is higher (as well as that of other economically important crops) (*SI Appendix*, Fig. S4). Importantly, our grid cell level results for cropland are statistically significant in contrast to our findings at the household level, due to the different power in the two regressions. The estimated cropland expansion represents 0.46% of the total grid cell area, which would imply an increase in cultivated land in the household data of 0.02 ha, an effect size that is consistent with our ITT estimate but which would require a much bigger sample than we have to detect.

Finally, we examine how preharvest maize prices are affected by FAW. In remote areas, where markets are incomplete, a negative supply shock will theoretically raise maize prices more than in a better-integrated market. Households may choose not to deforest if the profit from higher maize prices functions as a coping strategy; however, households may also capitalize on the higher maize price and expand the land in agricultural production. We use preharvest maize prices as our outcome variable, as this reflects when farmers observe the realization of their expected yield for a given year. We find that in high-FAW suitability grid cells (>80.7), maize prices increased by 2.75 to 6.48% on average after 2016 (*SI Appendix*, section and S1.7 Table S11, Panel *B*) compared to those with lower FAW suitability. However, when this effect is decomposed annually using an event study specification (*SI Appendix*, Table S11, Panel *A*), we find there are significant pretends and autocorrelation in maize prices (*SI Appendix*, Fig. S5, Models 1 and 2). In Model 3, we control for potential demand-side effects on maize prices and regional time trends. While our point estimate becomes smaller in magnitude (0.0275) and statistically insignificant in the DiD analysis, adding in these robust controls strengthens the event study results, providing suggestive evidence that maize prices increased in the years following the introduction FAW in Districts with high FAW suitability (*SI Appendix*, Fig. S5, Model 3).

## Discussion

Overall, we observe that FAW substantially decreased maize yields and increased food insecurity for smallholder farmers in Zambia. Food security is linked to FAW and charcoal production through a shock-response pathway: When FAW-induced crop failure undermines food availability, households often turn to charcoal production as a coping strategy to generate income and buffer the impact on their food security. Farmers that were more affected by FAW were likely to respond by either increasing charcoal production and/or increasing their cultivated acreage. Both responses are associated with deforestation. Specifically, we find that the introduction of FAW is associated with a 102% increase in deforestation (calculated at the median FAW suitability). To calculate how much of this increase in deforestation is driven by an increase in cropped area, we overlay the change in cropped area and find that 94% of the increase in cropped land happened in deforested land. This means that of the estimated effect of FAW on cropland expansion, 94% was associated with deforestation (1.93 ha of the estimated 2.06 ha), so that the FAW-driven expansion of cropland explains about 42% of the observed FAW-driven deforestation, or about 1.93 ha of the 4.6 ha of deforestation per grid cell. This finding is consistent with our observation that most of the FAW-driven deforestation happens in places with lower potential maize yield, while most of the FAW-driven cropland expansion happens in places with higher potential yield (*SI Appendix*, section 1.6 and Fig. S3). While we cannot directly attribute all other deforestation to charcoal, we do find that the household-level response of increasing the quantity of charcoal production by 22 to 40% could explain the rest. In summary, we observe both the Borlaug hypothesis—that a decrease in agricultural productivity increases farmland—and coping strategies are at play in driving the deforestation response to this negative agricultural productivity shock.

We observe that the largest increases in FAW-driven deforestation occur in places where there are both relatively accessible trees alongside relatively accessible markets for maize and charcoal. We also observe little deforestation and cropland expansion in locations with the wealthiest populations, suggesting that farmers in these locations have other coping strategies available to them other than those that require deforestation. This finding is consistent with the results from the household level analysis, where we find that the wealthiest households are less likely to produce charcoal in response to the arrival of the FAW and do not increase their cultivated land (*SI Appendix*, Fig. S2).

In other work (43), we show that households that have better access to FAW mitigation, such as pesticides, or greater linkages to urban areas through migration are less likely to resort to charcoal production as a coping strategy. This is also consistent with our findings for the wealthiest households, and for those who are closer to major towns (*SI Appendix*, Fig. S2), although we do not analyze whether migration to urban areas is the mediating mechanism. Hadunka (43) also shows that locations with greater crop diversity are less likely to resort to charcoal in response to a negative FAW shock. Thus, provision of alternative coping strategies might help mitigate the consumption of natural resources in response to a negative production shock. Further, the suggestion that improved yields might help reduce agricultural land expansion hints that other methods to increase farm productivity could mitigate against forest loss [as found in Malawi (28); Zambia (27), Uganda (8), and Brazil (29)]. On the other hand, our results suggest that as climate change delivers larger and more frequent negative production shocks, it might place more pressure on forests, increasing emissions and further exacerbating climate change.

Our analysis suffers from several data limitations. While we can observe deforestation and yields at a country-wide level, we do not have spatially granular data on charcoal production except from our household survey. Thus, we cannot explicitly say what fraction of deforestation is a result of increased charcoal production, but the fact that deforestation is more pronounced in regions that have greater access to urban areas while still having biomass is suggestive that charcoal might be the driver. Relatedly, we cannot evaluate whether charcoal remains a key coping mechanism beyond the first 3 y of the infestation. Second, we use Hansen et al. (17) data for deforestation, which is known to have limitations in capturing deforestation in woody savannahs (44)—which covers much of Zambia—as opposed to tropical forests, potentially biasing our results. That said, inasmuch as our analysis might miss deforestation in drier locations, FAW infestation levels were generally lower in the tropical forested areas. Thus, underestimates of deforestation in drier areas should, if anything, result in a downward bias of our estimates of FAW-induced deforestation. Third, our identification strategy relies on there being no other time-varying confounders that differentially affect locations that are more vs less susceptible to FAW infestation beyond our weather and yield controls. While the parallel pretrends give us confidence in our approach, and we find our results are robust to alternative specifications, we cannot definitively rule out other possible confounders.

In summary, our study finds that negative agricultural productivity shocks can lead to both agricultural land expansion and the consumption of forest products, both of which can lead to deforestation. Our research focuses on the FAW, but as climate change increases the threats of other insect pests along with increased weather challenges, our work suggests that climate change may pose additional pressures on forests, increasing emissions as it reduces agricultural productivity in developing countries.

## Materials and Methods

We use two broad approaches to estimate the effect of FAW. To estimate the effect of FAW on household outcomes, we use panel data on smallholder farming households from 10 districts across Zambia collected annually starting in June 2016 through August 2019 (summary statistics and map in *SI Appendix*, section S1.1). Because we observe household outcomes a year before FAWs arrived in Africa, we employ a differences-in-differences approach and test for significant differences between households who later are in infested areas versus not, both using summary statistics and controlling for leads (*SI Appendix*, section S1.3). We use village-level averages of other households’ reported measures of FAW infestation to control for potential reporting bias both as a direct measure and as an instrument for the household’s own reports. For food security, we use standard measures of dietary quality [food consumption score (FCS) and household dietary diversity score (HDDS)] as well as the reduced coping strategies index (rCSI) which captures the number of actions a household takes to cope with food shortages. More information on how these are derived is in *SI Appendix*, section S2.1. In all regressions, we use household and year fixed effects and weather controls. Details of the estimation methods and controls are discussed in the *SI Appendix*, section S3.1.

To estimate the effect that the arrival of the FAW had on deforestation, we use a machine learning (ML)-based approach. We use data before the arrival of the FAW to Zambia (pre-2017), to train a LASSO model that allows us to predict future deforestation for every year and every grid cell (*SI Appendix*, section S3.2). The predicted deforestation then represents a counterfactual of what deforestation would have been in the absence of the FAW, and thus the difference between the observed deforestation and the predicted deforestation (i.e. the “prediction error”) shows the ‘excess deforestation’ within each grid cell, as a consequence of the FAW. We then use this prediction error as our outcome variable to estimate how much of this counterfactual difference can be attributed to the FAW suitability after 2016. Note that this approach avoids the issue associated with two-way fixed effects estimation of multiperiod differences-in-differences in that our approach only ever uses untreated observations to generate the counterfactual (45).

The main reason for using the prediction error instead of the observed deforestation is that deforestation is a high-dimensional, nonlinear process. As such, controlling for this high-dimensionality and nonlinearity is easier to do in the context of a prediction model, where the explicit goal of the model is to closely approximate the data-generating process for deforestation before the arrival of the FAW. Given that the model is trained only using data up to 2016, the model’s predictions for 2017 onward are valid counterfactuals of what would have happened to deforestation within each grid cell in the absence of the FAW. If instead of the prediction error, we used the observed deforestation, our estimates of the effect that the FAW arrival had on deforestation could be biased (overestimated in some years and underestimated in others) if in our regression model we do not include all the factors that affect deforestation and are also potentially correlated with FAW suitability. The high dimensionality and nonlinearities associated with deforestation make this challenging in the context of a regression model, but less so in the context of a machine learning prediction model. In the latter, the use of cross-validation to select the explanatory variables that contribute more to the model’s out-of-sample predictive performance allows us to generate a counterfactual prediction of deforestation in the absence of the FAW, that takes into account all the relevant variables and possible nonlinearities in the data generation process. In addition to this, another source of bias comes from the fact that FAW suitability is likely to be correlated with variables that also affect deforestation (such as weather and other environmental conditions). Results using standard differences-in-differences approaches are given in the *SI Appendix*, section S1.6 and Table S10.

## Supplementary Material

Appendix 01 (PDF)

## Data Availability

All of the data documentation, data, and coding scripts necessary to replicate this project or reproduce the results of this paper have been deposited into this public GitHub repository: https://github.com/Protensia/PNAS---When-crops-fail-forests-follow.git.
