# Supplementary material for: When crops fail, forests follow: Agricultural shocks and deforestation in Zambia
Source: Proc Natl Acad Sci U S A. 2025 Oct 3;122(40):e2427156122. doi: 10.1073/pnas.2427156122 (PMC12519083; doi:10.1073/pnas.2427156122)
Supplement: Supplementary file 1 — Appendix 01 (PDF) [file pnas.2427156122.sapp.pdf]

PNAS

Supporting Information for

When crops fail, forests follow: Agricultural shocks and deforestation in Zambia

Pablo J. Ordonez, Protensia Hadunka, Gemma Del Rossi, Kathy Baylis

Pablo J. Ordonez
Email: pabloord@iadb.org

This PDF file includes:

Supporting text
Figures S1 to S6
Tables S1 to S11
SI References

S1. Results

S1.1 Summary statistics

Table S1. Summary statistics of household data at baseline.

Variable	Mean	S.D.	Min	Max
Age (years)	47.06	14.51	15	87
Gender (1 = male)	0.83	0.37	0	1
Education (years)	3.15	1.61	0	12
Household size	7.01	3.22	1	24
Charcoal (1 = yes)	0.2	0.4	0	1
Cultivated land (ha)	2.43	2.09	0.08	12
Total landholding (ha)	4.39	6.32	0	100
Maize yield (kg/ha)	1092.07	1191.79	0	20750
Total income (kwacha)	7530.71	15278.12	0	180000
Rainfall (mm)	949.06	150.66	727.86	1306.18
Temperature	33.57	3.45	27.24	40.97
Access to credit (1 = yes)	0.72	0.45	0	1
Distance to the forest (km)	7.3	5.99	0	40
Food Consumption Score (FCS)	2.8	0.47	1	3
Household Dietary Diversity Score (HDDS)	1.26	0.5	1	3
Reduced Coping Strategies Index (rCSI)	2.02	1.00	1	3
Observations at baseline	745			

Notes: Table S1 summarizes survey baseline household (balanced panel) and food security characteristics.

Table S2. Summary statistics of gridcell data.

	Mean	S.D.	Min	Max
Constant				
FAW Suitability (0-100)	72.2	19.1	6.7	88.3
Tree cover in 2000 (%)	24.9	15	0	75.8
Grid cell area (ha)	477.9	12.8	129.7	487.4
Travel time to closes town with >50k pop.	607.6	432	1.1	2,821.20
Elevation (mts)	1,120.20	220.7	329.4	2,235.80
Yield potential (kg/ha) - Maize	3,298.50	1,199.00	0	7,251.00
Yield potential (kg/ha) - Soybean	1,382.40	392.3	0	2,719.00
Yield potential (kg/ha) - Cassava	499.4	208.9	0	996.3
Yield potential (kg/ha) - Sugarcane	801.4	726.5	0	2,619.00
Yield potential (kg/ha) - Groundnut	789.4	275.1	0	1,642.00
Total number of grid cells	158,469			
Average for 2012-2016				
Deforestation (Ha)	1.5	5.2	0.0	467.6
Yearly Rainfall (mm)	953.2	212.9	398.2	1847.3
Yearly Temperature (Celsius)	30.5	2.7	20.8	39.0
Population	417.4	2474.8	0.0	185,548

Notes: Table S2 summarizes the characteristics of the gridcells used for the machine learning models.

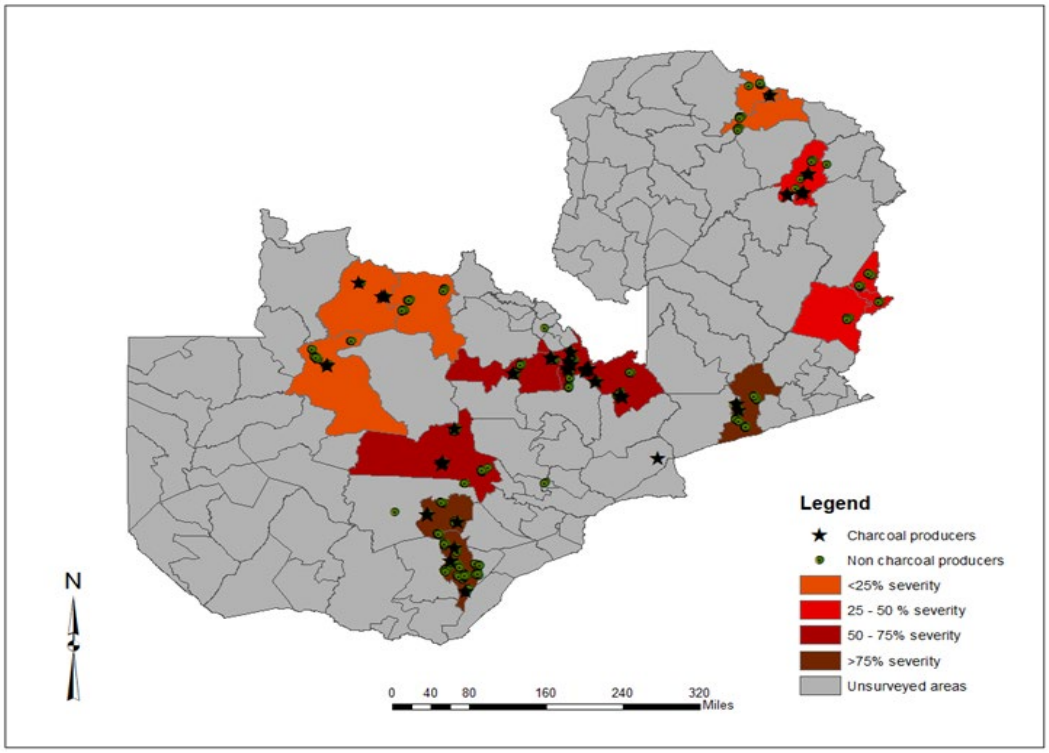

Figure S1. Districts and locations of surveyed households. The red shaded areas represent the districts that were surveyed during primary data collection.

S1.2 Full OLS, ITT and LATE results for primary household data

Table S3. Effects of FAW on maize yields and food security outcomes.

	(1)	(2)	(3)	(4)	(5)	(6)	(7)	(8)	(9)	(10)	(11)	(12)
**VARIABLES**	**Log(Yield)**			**ihs(FCS)**			**ihs(HDD)**			**ihs(rCSI)**		
	**OLS**	**ITT**	**LATE**	**OLS**	**ITT**	**LATE**	**OLS**	**ITT**	**LATE**	**OLS**	**ITT**	**LATE**
FAW	-0.023	-0.113**	-0.349**	-0.002	-0.041***	-0.071***	-0.018***	-0.069***	-0.122***	0.288***	0.620***	0.344***
	(0.022)	(0.046)	(0.148)	(0.006)	(0.009)	(0.015)	(0.0093)	(0.009)	(0.015)	(0.012)	(0.016)	(0.016)
Controls	Yes	Yes	Yes	Yes	Yes	Yes	Yes	Yes	Yes	Yes	Yes	Yes
HHFE	Yes	Yes	Yes	Yes	Yes	Yes	Yes	Yes	Yes	Yes	Yes	Yes
Year FE	Yes	Yes	Yes	Yes	Yes	Yes	Yes	Yes	Yes	Yes	Yes	Yes
Observations	2,732	2,732	2,732	2,980	2,767	2,767	2,980	2,767	2,767	2,980	2,767	2,767

Notes: Regressions analyze household self-reported FAW infestation (OLS), village average infestation levels (ITT), or farmer-level infestation instrumented by village averages (LATE). All models control for rainfall (mm), temperature (in degrees celsius), and their squares during the growing season, with household and year fixed effects. Maize yield regressions include input controls, and LATE estimates rely on strong instruments for self-reported FAW incidence (see the first stage results in Table S4 below). Yields are log-transformed, and all other outcomes are transformed using the inverse hyperbolic sine (IHS) transformation. The maize yields are estimated using linear fixed effects models, and the food security and wellbeing measures (FCS, HDD, rCSI) are estimated using Tobit models with random effects. All regressions used standard errors clustered at the camp level. Statistical significance is denoted as *** $p < 0.01$, ** $p < 0.05$, and * $p < 0.1$.

Table S4. First stage of the effects of FAW on maize yields and food security outcomes.

Variable	Instrument	Coefficient	Standard Error	t-Statistic	p-Value	F-Statistic
Log(Yield)	Average FAW at camp level	0.322	0.060	5.91	0.000	29.13
ihs(FCS)	Average FAW at camp level	0.089	0.017	4.2	0.000	81.8
ihs(HDD)	Average FAW at camp level	0.131	0.017	7.92	0.000	15.84
ihs(rCSI)	Average FAW at camp level	0.561	0.059	18.54	0.000	28.61

Notes: The average FAW at the camp level serves as a strong and significant instrument across all variables in the table. For yield, the coefficient is 0.3224 with a standard error of 0.0597, resulting in a t-statistic of 5.91 (p-value = 0.000) and an F-statistic of 29.13, demonstrating moderate instrument strength. For FCS, the coefficient is 0.0892 with a standard error of 0.01670, yielding a significant t-statistic of 4.2 (p-value = 0.000) and an F-statistic of 81.8, indicating strong instrument relevance. Similarly, for HDD, the coefficient is 0.1314 with a standard error of 0.0166, resulting in a t-statistic of 7.92 (p-value = 0.000) and an F-statistic of 15.84, again reflecting this is a suitable instrument. Finally, for rCSI, the coefficient is 0.5615 with a standard error of 0.0586, producing a large t-statistic of 18.54 (p-value = 0.000) and an F-statistic of 28.61, confirming strong instrument relevance for this variable as well.

Table S5. Effects of FAW on charcoal and cultivated land in the following year.

	(1)	(2)	(3)	(4)	(5)	(6)	(7)	(8)	(9)
**VARIABLES**	**Charcoal (1 = Yes)**			**Ihs(Charcoal (Quantity produced))**			**log(Cultivated land (ha))**		
	**OLS**	**ITT**	**LATE**	**OLS**	**ITT**	**LATE**	**OLS**	**ITT**	**LATE**
FAW	0.026*	0.039***	0.0356***	0.085	0.316**	0.165**	-0.017	0.030	0.083
	(0.008)	(0.013)	(0.015)	(0.065)	(0.156)	(0.086)	(0.019)	(0.043)	(0.115)
Controls	Yes	Yes	Yes	Yes	Yes	Yes	Yes	Yes	Yes
HH FE	Yes	Yes	Yes	Yes	Yes	Yes	Yes	Yes	Yes
Year FE	Yes	Yes	Yes	Yes	Yes	Yes	Yes	Yes	Yes
Observations	1,943	1,943	1,943	1,572	1,572	1,572	2,234	2,039	2,039

Notes: Regressions examine self-reported FAW infestation (OLS), average infestation (ITT), and farmer-level infestation instrumented by village averages (LATE). Charcoal production probability (binary: 1 = produced, 0 = did not produce) is modeled using a CRE probit, while charcoal quantity uses a Tobit random effects model, and cultivated land regressions use a linear two-way fixed effects model. LATE estimates rely on strong instruments for self-reported FAW incidence (see first-stage results). All models include controls for rainfall (mm), temperature (in degrees Celsius), and their squares, with household and year-fixed effects to address unobserved heterogeneity and temporal variation. In addition to the weather controls, the yield model also uses the cultivated land as a control variable. The quantity of charcoal produced is transformed using the inverse hyperbolic sine (IHS) transformation while cultivated land is transformed using a logarithmic transformation. All regressions used standard errors clustered at the camp level. Statistical significance is denoted as *** $p < 0.01$, ** $p < 0.05$, and * $p < 0.1$.

Table S6. First stage results of the effects of FAW on charcoal and cultivated land in the following year.

Variable	Instrument	Coefficient	Standard Error	t-Statistic	p-Value	F-Statistic
Charcoal (1 = Yes)	Lag Average FAW at camp level	0.608	0.357	7.68	0.000	59.06
ihs(Quantity charcoal)	Lag Average FAW at camp level	0.621	0.468	1.33	0.190	1.76
log(Cultivated land)	Lag Average FAW at camp level	0.411	0.067	6.1	0.000	7.25

Notes: The lagged average FAW at the camp level proves to be a strong and significant instrument for charcoal production probability and cultivated land but weaker for quantity charcoal produced. For charcoal production probability, the coefficient is 0.6076 with a standard error of 0.3568, yielding a t-statistic of 7.68 (p-value = 0.000) and a large F-statistic of 59.06, indicating strong instrument relevance. Similarly, for cultivated land, the coefficient is 0.4106 with a standard error of 0.0673, resulting in a t-statistic of 6.1 (p-value = 0.000) and an F-statistic of 7.25, suggesting moderate instrument strength. However, when estimating the effect of FAW on the inverse hyperbolic sine of the quantity charcoal, the coefficient of the average level of FAW on self-reported FAW is 0.6212 with a standard error of 0.4679, producing a t-statistic of 1.33 and a non-significant p-value of 0.190, with an F-statistic of 1.76, indicating weak instrument relevance for this variable.

S1.3 Leads regressions for primary data

Table S7. Test of leads on the effects of FAW on maize yields and food security outcomes.

	(1)	(2)	(3)	(4)	(5)	(6)	(7)	(8)	(9)	(10)	(11)	(12)
**VARIABLES**	**ihs(Yield)**			**ihs(FCS)**			**ihs(HDD)**			**ihs(rCSI)**		
	**OLS**	**ITT**	**LATE**	**OLS**	**ITT**	**LATE**	**OLS**	**ITT**	**LATE**	**OLS**	**ITT**	**LATE**
FAW	-0.034	-0.183**	-0.444**	-0.011	-0.037***	-0.056***	-0.026***	-0.069***	-0.101***	0.349***	0.652***	0.887***
	(0.037)	(0.075)	(0.191)	(0.007)	(0.010)	(0.013)	(0.006)	(0.008)	(0.012)	(0.014)	(0.015)	(0.026)
Lead FAW	0.034	-0.037	0.015	-0.016	-0.021*	-0.018*	-0.027	-0.036*	0.0315	-0.012	-0.037	0.194**
	(0.062)	(0.067)	(0.287)	(0.012)	(0.013)	(0.008)	(0.011)	(0.011)	(0.031)	(0.026)	(0.223)	(0.019)
Controls	Yes	Yes	Yes	Yes	Yes	Yes	Yes	Yes	Yes	Yes	Yes	Yes
HHFE	Yes	Yes	Yes	Yes	Yes	Yes	Yes	Yes	Yes	Yes	Yes	Yes
Year FE	Yes	Yes	Yes	Yes	Yes	Yes	Yes	Yes	Yes	Yes	Yes	Yes
Observations	1,915	1,767	1,767	2,072	1,915	1,985	2,072	1,985	1,985	2,072	1,985	1,985

Notes: The results indicate that the effects of Lead FAW are generally small in magnitude and not statistically significant for yield and household dietary diversity, and slightly significant ($p < 0.1$) for food consumption scores (FCS) and reduced coping strategies index (rCSI) ($p < 0.05$). This suggests that the primary impact of FAW for certain outcomes is contemporaneous, meaning that current FAW infestations have immediate effects on yield and household welfare, rather than being anticipated by households in advance. All regressions used standard errors clustered at the camp level. Statistical significance is denoted as *** $p < 0.01$, ** $p < 0.05$, and * $p < 0.1$.

Table S8. Test of leads on the effects of FAW on charcoal and cultivated land in the following year.

	(1)	(2)	(3)	(4)	(5)	(6)	(7)	(8)	(9)
**VARIABLES**	Charcoal (1 = Yes)			ihs(Charcoal (Quantity produced))			log(Cultivated land (ha))		
	**OLS**	**ITT**	**LATE**	**OLS**	**ITT**	**LATE**	**OLS**	**ITT**	**LATE**
Lag FAW	0.031***	0.040**	0.0351***	0.089	0.2162**	0.167	-0.020	0.057	0.139
	(0.012)	(0.022)	(0.0053)	(0.066)	(0.1114)	(0.085)	(0.020)	(0.048)	(0.121)
Lead	-0.017**	-0.081*	0.0042	-0.040	0.0025	-0.056	-0.007	0.010	-0.001
	(0.010)	(0.061)	(0.0032)	(0.067)	(0.0131)	(0.071)	(0.016)	(0.039)	(0.157)
Controls	Yes	Yes	Yes	Yes	Yes	Yes	Yes	Yes	Yes
HH FE	No	No	No	Yes	Yes	Yes	Yes	Yes	Yes
Year FE	No	No	No	Yes	Yes	Yes	Yes	Yes	Yes
Observations	1,781	1,653	1,653	1,572	1,572	1,572	2,162	1,898	1,898

Notes: The results show that Lead FAW, representing anticipated current infestations, has no significant impact on charcoal production, quantity produced, or cultivated land. Households do not adjust their production or land-use decisions in response to future FAW, indicating their responses are driven by current or past infestations rather than anticipation. All regressions used standard errors clustered at the camp level. Statistical significance is denoted as *** $p < 0.01$, ** $p < 0.05$, and * $p < 0.1$.

S1.4 Heterogeneity analysis using household level data

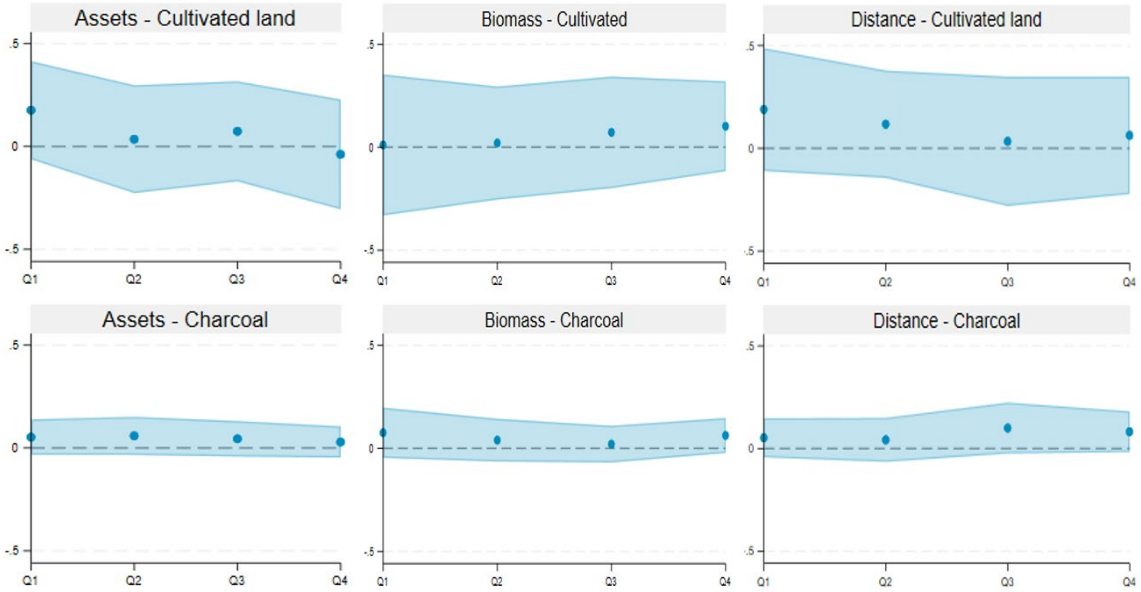

Figure S2. Heterogeneity analysis on the cultivated land and charcoal production response to the FAW. The categories we conducted the heterogeneity analysis are: household assets (turned into an asset index), mean biomass (estimated as a 5km buffer around each household), and distance to town center as mean across the camps (distance from the center of the camp to town center). Each variable is with respect to 2016 (baseline) levels – if we were to include later years, there would likely be unaccounted for interactions with FAW driving the effect on the outcomes of interest. The points are the estimated treatment effect by quartile of each variable's distribution. The light blue bands are the 95% CI for each point estimate. The outcome variables are log(cultivated land(ha)) and a binary charcoal production variable (estimated with CRE probit), and the estimated coefficients come from the interaction of each variable's instruments from Table S5, and a factor variable with four values (1 to 4) that denote the quartile for each household for the variable of interest. The variables were derived using data from (1) for assets and distance to town centers, and (2) for biomass measurements.

S1.5 Machine learning results

We compare the performance of the ML models for predicting both deforestation and deforestation, against a benchmark model based on an OLS regression that includes the same covariates included in our ML models. We find that for yields, at the district level (Table S9, Panel A), the mean prediction from the OLS model is significantly higher than the ground truth (8 ton/ha), and the standard deviation is also significantly higher, which translates into a much higher RMSE for the OLS model than the ML model. The ML model results in a mean predicted yield that is close to the ground truth and as such, the RMSE that results from the ML model prediction is 61% lower than the one from the OLS model. For deforestation, the gain in prediction accuracy is more modest. The mean predicted deforestation from the OLS model is higher than the ground truth, compared to the mean predicted deforestation from the ML model. However, the predicted deforestation from the OLS models has a higher dispersion, which better approximates some of the dispersion in the ground truth dispersion, which results in a modest reduction in the RMSE from going from the OLS model to the ML model (Table S9, Panel B).

Table S9. Machine Learning and OLS Models Performance (validation sample).

**Panel A.** ML Model for yield prediction		
	Mean	SD
Observed maize yield (Ton/Ha)	2.363	1.303
OLS prediction (Ton/Ha)	7.997	11.170
OLS Model RMSE	2.344	-
Lasso prediction	2.402	1.097
Lasso Model RMSE	0.906	-
% for RMSE	-61.3%	

**Panel B.** ML for deforestation prediction		
	Mean	SD
Deforestation (ha)	1.39	5.080
OLS prediction (ha)	2.035	2.493
OLS Model RMSE	4.965	
Lasso prediction (ha)	1.359	1.161
Lasso Model RMSE	4.889	
% for RMSE	-1.5%	

S1.6 Regression analysis on prediction errors

To estimate the effect that the arrival of the FAW had on deforestation, we estimate two types of models. In both cases, we use two dependent variables: the prediction error in deforestation (observed deforestation minus predicted deforestation) and the observed deforestation. One set of models is based on event study regressions, which allow us to recover evidence about the existence of pre-trends in deforestation associated with the level of FAW suitability. We find that prior to the arrival of the FAW, there is no difference in the level of deforestation associated with different levels of FAW suitability, which we believe provides evidence that supports the validity of the parallel trends assumption (Table S10, Panel A). The second set of models is based on a differences-in-differences specification, where we interact a post FAW dummy (which is equal to 1 for all years after 2016) with FAW suitability index (Table S10, Panel B). From these set us results, it is important to highlight how the results from the models that use the observed deforestation instead of the prediction error for deforestation, result in a higher estimated effect from the arrival of the FAW. For our preferred specification (Model 4), the estimated effect with the observed deforestation, are 17% higher than the estimated effect when using the prediction error as our outcome variable.

Table S10. Regression results for deforestation analysis at grid cell level.

Panel A. Event study regressions

	Dependent variable: Prediction error (ha) (Standard errors in parentheses)				Dependent variable: Deforestation(ha) (Standard errors in parentheses)			
	Model 1	Model 2	Model 3	Model 4	Model 1	Model 2	Model 3	Model 4
2012 x FAW Suitability	0.002	0.004	0.001	0.004	-0.001	-0.002	-0.002	-0.002
	(0.021)	(0.024)	(0.021)	(0.024)	(0.021)	(0.024)	(0.021)	(0.024)
2013 x FAW Suitability	-0.009	-0.011	-0.009	-0.011	-0.011	-0.012	-0.011	-0.012
	(0.020)	(0.022)	(0.020)	(0.022)	(0.020)	(0.023)	(0.020)	(0.023)
2014 x FAW Suitability	0.006	0.004	0.006	0.004	0.005	0.003	0.005	0.002
	(0.021)	(0.023)	(0.021)	(0.023)	(0.020)	(0.022)	(0.021)	(0.022)
2015 x FAW Suitability	-0.002	-0.002	-0.002	-0.002	-0.006	-0.007	-0.006	-0.007
	(0.019)	(0.021)	(0.020)	(0.021)	(0.019)	(0.021)	(0.020)	(0.021)
2017 x FAW Suitability	0.021**	0.023**	0.021**	0.023**	0.019	0.021	0.019	0.021
	(0.019)	(0.022)	(0.020)	(0.022)	(0.019)	(0.022)	(0.020)	(0.022)
2018 x FAW Suitability	0.013	0.019	0.013	0.019	0.018	0.023	0.018	0.023
	(0.020)	(0.022)	(0.021)	(0.023)	(0.020)	(0.023)	(0.020)	(0.023)
2019 x FAW Suitability	0.009	0.011	0.010	0.011	0.011	0.013	0.013	0.013
	(0.021)	(0.025)	(0.022)	(0.025)	(0.019)	(0.023)	(0.020)	(0.023)
Proxy for maize yield	No	No	Yield (ton/ha)	Yield (ton/ha)	No	No	Yield (ton/ha)	Yield (ton/ha)
Weather var. (t and t-1)	No	Yes	No	Yes	No	Yes	No	Yes
Observations	10,107	10,107	10,107	10,107	10,107	10,107	10,107	10,107

Panel B. Difference-in-Difference estimation

	Dependent variable: Prediction error (ha) (Standard errors in parentheses)				Dependent variable: Deforestation(ha) (Standard errors in parentheses)			
	Model 1	Model 2	Model 3	Model 4	Model 1	Model 2	Model 3	Model 4
Post x Suitability	0.020***	0.019***	0.020***	0.019***	0.023***	0.022***	0.023***	0.022***
	(0.010)	(0.012)	(0.010)	(0.012)	(0.010)	(0.012)	(0.010)	(0.012)
Proxy for maize yield	No	No	Yield (ton/ha)	Yield (ton/ha)	No	No	Yield (ton/ha)	Yield (ton/ha)
Weather var. (t and t-1)	No	Yes	No	Yes	No	Yes	No	Yes
Observations	10,107	10,107	10,107	10,107	10,107	10,107	10,107	10,107

Notes: Panel A includes the results from the event study regressions, and Panel B includes the results from a difference-in-difference specification. In both cases, the covariates and fixed effects included vary by model. Model 1 includes no covariates. Model 2 includes mean rain and temperature by grid cell, both contemporaneous and lagged one year. Model 3 includes the predicted maize yield per grid cell and year. Model 4 includes the predicted maize yield, as well as mean rain and temperature (both contemporaneous and lagged one year). Our preferred specification is Model 4, and this is the one that we use for the heterogeneity analysis.

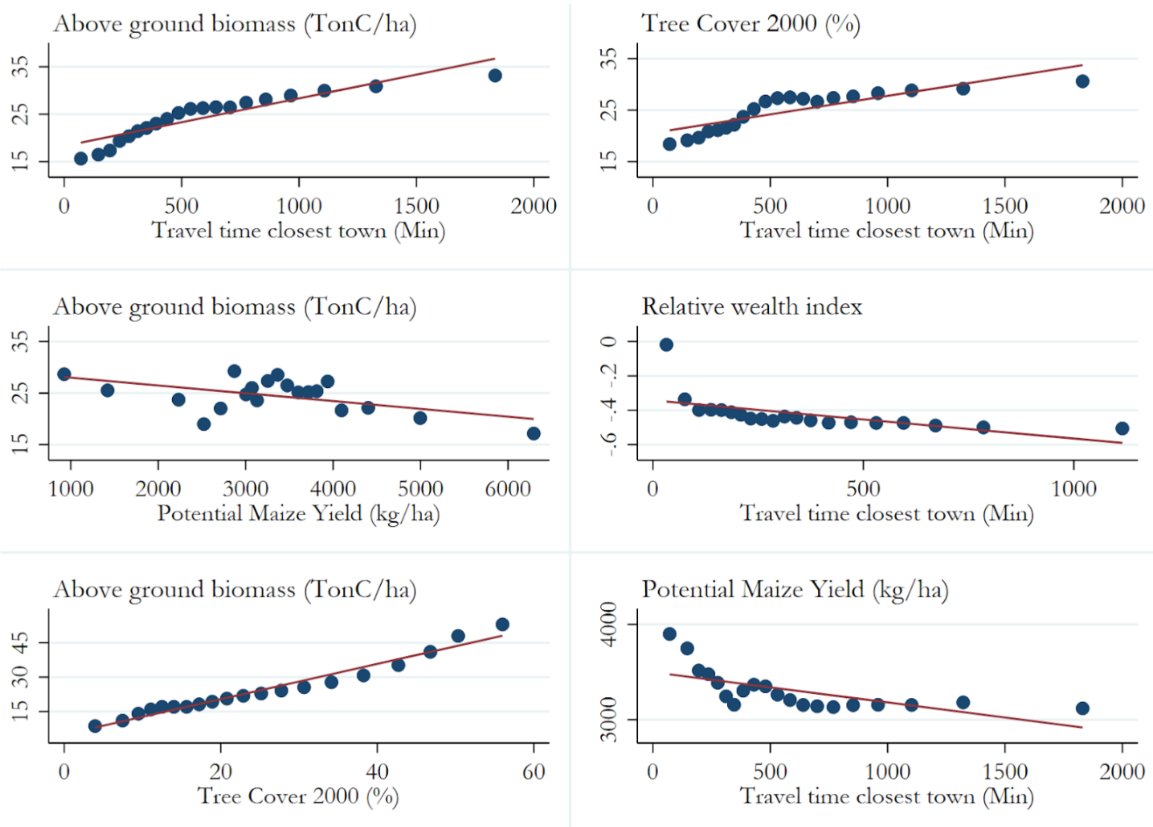

Figure S3. Bin-scatter plots of characteristics for heterogeneity analysis.

Panel A. Effects on FAW on deforestation by potential maize yields

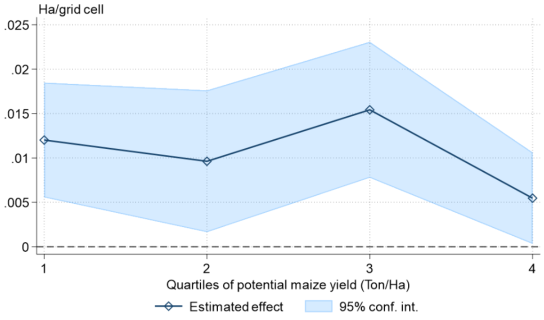

Panel B. Effects on FAW on deforestation by potential maize yields

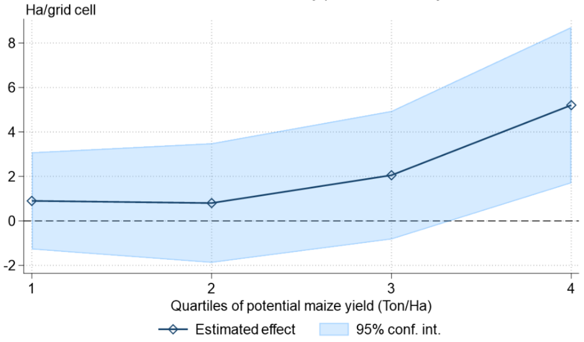

Panel C. Mean of main variables by the quartiles of potential maize yields

Quartiles of potential maize yield	Potential maize yield	Potential soybean yield	Potential sorghum yield	Potential cassava yield	Travel time to closest town	Percentage tree cover	FAW suitability index
	ton/ha	ton/ha	ton/ha	ton/ha	minutes	% of grid cell	
1	1,860	1,008	1,537	349	645	22	75
2	3,049	1,227	2,470	436	725	25	74
3	3,584	1,496	2,909	650	580	31	71
4	4,733	1,807	3,717	564	484	22	69

Figure S4. Deforestation and cropland heterogeneity analysis by potential maize yield.

S1.7 Results on maize prices and FAW suitability

Table S11. Regression results for pre-harvest maize prices at the gridcell level

Panel A. Event study regressions

	Dependent variable: Mean maize price, pre-harvest (Standard errors in parentheses)		
	Model 1	Model 2	Model 3
2010 x Treatment = 1	-0.0981*	-0.0506	-
	(0.0545)	(0.0527)	
2011 x Treatment = 1	-0.0951*	-0.0574	-0.0082
	(0.0482)	(0.0430)	(0.0331)
2012 x Treatment = 1	-0.0428	-0.0110	-0.0009
	(0.0365)	(0.0415)	(0.0319)
2013 x Treatment = 1	-0.0863	-0.0633	0.0077
	(0.0593)	(0.0583)	(0.0209)
2014 x Treatment = 1	-0.0635	-0.0515	-0.0051
	(0.0493)	(0.0415)	(0.0262)
2015 x Treatment = 1	-0.0198	0.0127	0.0420
	(0.0523)	(0.0524)	(0.0282)
2017 x Treatment = 1	-0.0264	0.0164	-0.0093
	(0.0292)	(0.0311)	(0.0313)
2018 x Treatment = 1	0.0390	0.0579	0.0397
	(0.0590)	(0.0602)	(0.0317)
2019 x Treatment = 1	0.0268	0.0359	0.0786**
	(0.0483)	(0.0494)	(0.0320)
Predicted maize yield	No	Yes	Yes
Weather in t-1	No	Yes	Yes
Demand controls	No	No	Yes
Year FE	Yes	Yes	Yes
Grid cell FE	Yes	Yes	Yes
Province-Year FE	No	No	Yes
Observations	820,357	805,552	699,863

**Panel B.** Difference-in-Difference Estimation			
	Dependent variable: Mean maize price, pre-harvest (Standard errors in parentheses)		
	Model 1	Model 2	Model 3
Post x Treatment = 1	0.0640***	0.0648***	0.0275
	(0.0167)	(0.0168)	(0.0199)
Predicted maize yield	No	Yes	Yes
Weather in t-1	No	Yes	Yes
Demand controls	No	No	Yes
Year FE	Yes	Yes	Yes
Grid cell FE	Yes	Yes	Yes
Province-Year FE	No	No	Yes
Observations	820,357	805,552	699,863

Notes: Panel A includes the results from the event study regressions, and Panel B includes the results from a difference-in-difference specification. The outcome variable is annual District-level maize prices, averaged across the pre-harvest months (February, March, and April). The treatment variable is defined as gridcells that have a higher-than median suitability (>80.7), and therefore untreated gridcells are those with FAW suitability < 80.7. In both analyses, the covariates and fixed effects included vary by model. Model 1 includes no covariates. Model 2 includes monthly mean rain and temperature by grid cell lagged one year and predicted maize yield per grid cell and year to mirror the specifications for FAW on deforestation and cropland expansion. Model 3 includes demand-side drivers of maize prices as controls, including mean pre-harvest maize price lagged one year and annual district population, and Province-Year fixed effects to control for region-wide time trends.

Panel A. Event study results of FAW on district maize prices, pre-harvest.

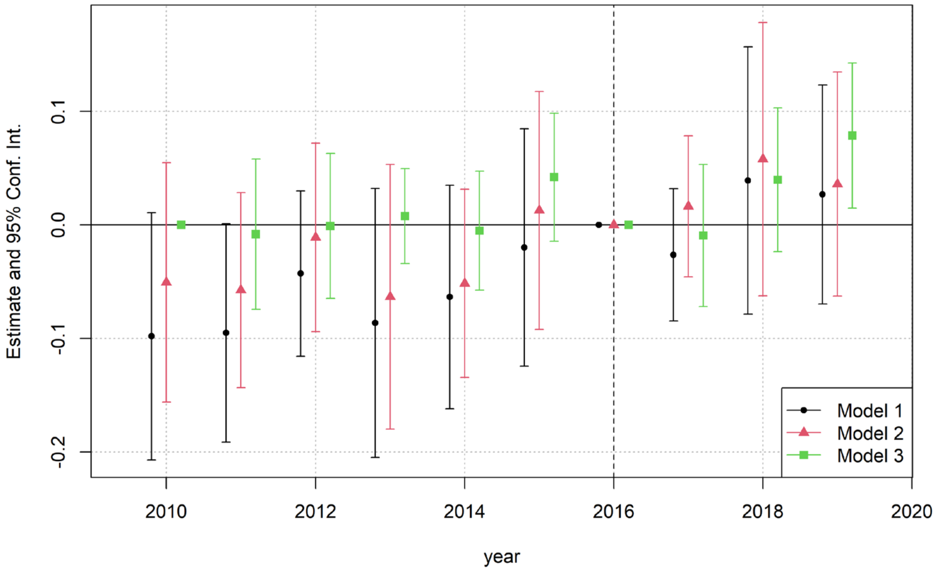

Panel B. Difference-in-Differences results of FAW on district maize prices, pre-harvest.

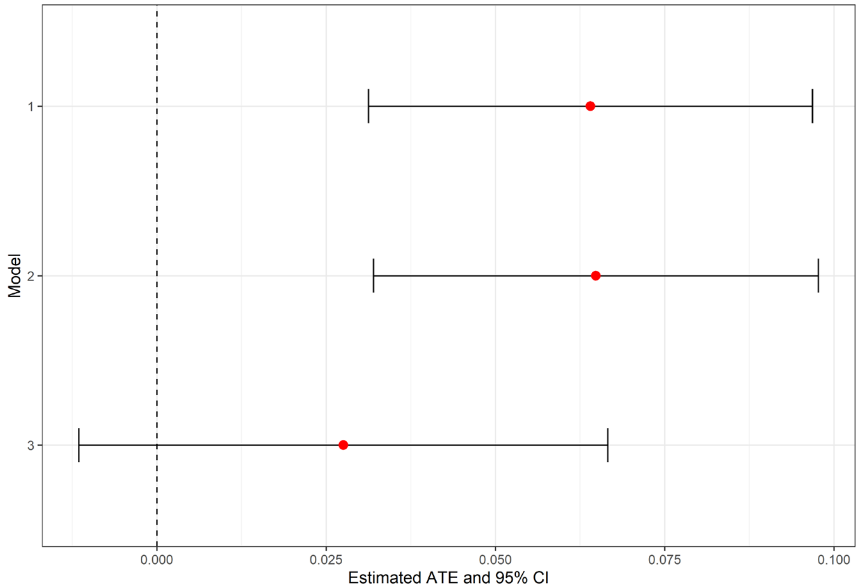

Figure S5. Event study and difference-in-differences results on the impact of FAW arrival on district pre-harvest maize prices. We find that on average, maize prices increased in gridcells that had higher FAW suitability. Each point represents an estimated treatment effect found in Table S11, and the bars represent the 95% confidence interval for each given point estimate.

S2. Data

S2.1 Food security and household welfare measures

Food Consumption Score (FCS)

The Food Consumption Score (FCS) is a composite score developed by the World Food Programme (WFP) that reflects dietary diversity, food frequency, and the relative nutritional importance of different food groups. It is calculated based on the number of days specific food groups were consumed by a household over a 7-day recall period. Each food group is assigned a weight based on its nutritional value, and the weighted frequencies are summed to generate the total FCS.

According to the WFP's 'Indicators and Food Security and Nutrition Integration Guide'[1], the FCS is categorized into three food consumption groups. These categories are used to classify households' food security status and inform programmatic decisions:

**FCS Range**	**Category**
FCS ≤ 21	Poor
21 < FCS ≤ 35	Borderline
FCS > 35	Acceptable

These thresholds assume occasional or irregular consumption of oil and sugar.

Reduced Coping Strategies Index (rCSI) Categorization

The Reduced Coping Strategies Index (rCSI) measures the frequency and severity of five common food-related coping strategies over the past 7 days. Each strategy is assigned a severity weight based on guidance from the WFP Coping Strategies Index Manual[2].

**Strategy**	**Severity Weight**
1. Rely on less-preferred, less-expensive foods	1
2. Borrow food or help from friends/relatives	2
3. Limit portion size at mealtimes	1
4. Restrict adult consumption so children can eat	3
5. Reduce number of meals	1

After summing the weighted frequencies, the total rCSI score were categorized as follows:

**rCSI Score**	**Category**
0–3	Low coping
4–18	Medium coping
>18	High coping

Household Dietary Diversity Score (HDDS) Categorization

[1] Available at: https://www.dropbox.com/s/dopgy5moow6kf64/5.WFP_IndicatorsFSandNutIntegration.pdf?dl=0
[2] To see references on standard strategies and severity weights:
https://www.ennonline.net/attachments/906/coping-strategies-index-manual-second-edition-(final)[1].pdf
http://www.securenutrition.org/sites/default/files/resources/attachment/english/maxwell-d_2013_how-do-different-indicators-of-hfs-compare.pdf
http://www.fao.org/fileadmin/user_upload/food-security-capacity-building/docs/Nutrition/NairobiWorkshop/5.WFP_IndicatorsFSandNutIntegration.pdf

The Household Dietary Diversity Score (HDDS) is a measure of the number of different food groups consumed by a household over the past 24 hours. It is used as a proxy indicator for the economic ability of a household to access a variety of foods. The HDDS is calculated by summing the number of distinct food groups consumed from a predefined list (typically 12 groups). Each food group consumed receives a score of 1, regardless of quantity or frequency, and the total score ranges from 0 to 12.

Based on thresholds suggested by Vaitla et al. (3) in “Comparing Household Food Consumption Indicators to Inform Acute Food Insecurity Phase Classification”, HDDS scores are grouped into three categories to classify the food security status of a household[3]:

HDDS Score	Category
0–3	Severely food insecure
4–5	Moderately food insecure
6–12	Food secure / Mildly food insecure

S2.2 Gridded machine learning data

The data we use come from different sources, and we combine them into a single dataset, by creating a 0.02 x 0.02 degrees (around 4.41 sq. km) gridded dataset that covers all of Zambia. For each grid cell, we have the total deforestation by year, as well as the rainfall, temperature and the normalized difference vegetation index (NDVI) by year and month, the potential yield for a set of thirteen crops, the elevation, travel distance to main towns, and the estimated population.

The deforestation data we use come from a publicly available dataset of global forest loss developed by Hansen et al. (4). They use Landsat 7 ETM images to construct a worldwide dataset of forest cover and loss between 2001 and 2019 (version 1.6 reprocessed the data since 2011, using Landsat 8 OLI data). It is important to note that the 1.6 release of the data has a processing method that is different from previous versions. Therefore, they caution about the compatibility of the data for the period 2001-2012, and data for the latest version (2011-2019). It is also important to note that the environmental characteristics of the wooded regions in Zambia, where most of the land can be classified as woodlands (miombo woodlands being the predominant vegetation type), can potentially lead to an overestimation of the forest area and an underestimation of deforestation, with areas with low woody cover being the ones where underestimation is more prevalent (5). However, given the availability of Hansen’s deforestation data as a consistent time series for our study region, we will use these data as our measure of deforestation, and we believe that any effects we find will be a lower bound of the true effects on deforestation.

Additionally, to capture the presence of the FAW, we use the data generated by Early et al. (6). They use an ensemble species distribution model, together with the reported presence of the FAW, to train a model that can predict the relative suitability of year-round presence of the FAW in a given region, based on the land use in that region and climatic variables. The index they create ranges from 0 to 1, and so a region with a value of 0.6 is twice as suitable as one with a value of 0.3. The model does not predict dynamic changes, so it does not tell us how suitability changes with changes in weather and land use from one period to the next.

We also include weather data, mainly rainfall and temperature. The rainfall data, from the Climate Hazards Group Infrared Precipitation with Stations (CHIRPS) dataset, which is built around a 0.05° climatology using satellite information, with data at a daily, pentadal, monthly, and yearly frequency, from 1981 to the present day (7). We will use the monthly data to capture seasonal effects on the crops’ yields. The land temperature and NDVI data come from the Terra satellite Moderate Resolution Imaging Spectroradiometer (MODIS), with a resolution of 0.05 degrees, which means that each pixel covers an area of 26.01 sq. km (8, 9). The NDVI data is an index that captures the

[3] For more: https://www.dropbox.com/s/dopgy5moow6kf64/5.WFP_IndicatorsFSandNutIntegration.pdf?dl=0

degree of ‘greenness’ within a grid cell, with higher values indicating that there are more leaves from trees, shrubs and crops, and lower values indicating the opposite.

The two additional variables included are the population and potential yield for different crops. The population data come from the gridded population dataset, which is a raster of the estimated population per pixel, with a resolution of 2.5 arc-minutes (approximately 4.63 km) for the years 2000, 2005, 2010, 2015, and 2020 (10). To have data for the other years, we assume a constant rate of change between the years with data. For example, we calculate the yearly growth rate for the years between 2000 and 2005, so that for a given grid cell, the imputed value for 2001 would be the value in 2000 multiplied by the estimated change rate. The potential yield data come from the global agro-ecological zones dataset, which models the estimated potential yield for each crop based on the weather and soil characteristics in a given region (11).

Additionally, to understand how the FAW affected maize production, and the ability that the FAW suitability index has in predicting those effects, we use two different panel datasets. The first one is the Crop Forecast Survey (CFS), which is an annual survey of small and medium holding farmers, done by the Central Statistical Office (12). For our analysis, we use the data aggregated at the district level (72 districts) on total area planted and harvested, the expected production and the yield. The other dataset that we use is a panel of smallholder farmers called the Household Income, Consumption, and Production Survey (HICPS), conducted in 2016, 2017, 2018, and 2019 (1). This survey covered the 2015/16, 2016/17, 2017/18 and 2018/19 agricultural seasons, with approximately 742 smallholder households surveyed in 12 districts of Zambia and data collected on socioeconomic and demographic characteristics, production activities, income sources, and insect pest infestation.

S3. Methods

S3.1 Methods used for the primary data analysis

To analyze the binary outcome of household participation in charcoal production, we use a correlated random effects (CRE) probit model to estimate the impact of FAW. While the standard panel probit model assumes independence between covariates and the time-invariant error term, this assumption may not always hold. To address potential correlation, we apply the Mundlak device, which models the time-invariant error as a function of the means of time-varying covariates. This approach adjusts the CRE probit coefficients to account for the time-invariant error and avoids the incidental parameter problem commonly found in fixed effects estimations for nonlinear models. Consequently, the CRE probit model with the Mundlak device is a superior choice for nonlinear panel data analysis compared to linear fixed effects models (13, 14).

For the full sample, we use a CRE model to account for the unbalanced panel data structure and the non-linear nature of the binary dependent variable, which equals 1 if the household participates in charcoal or firewood production and 0 otherwise. In the first year, the dependent variable is binary, but in subsequent years, it transitions to a continuous measure of charcoal production quantity. The model is specified as follows:

$$P(y_t = 1 \mid x_{it}, FAW_{it-1}) = \Phi\big(\beta FAW_{it-1} + x'_{it}\gamma + \underline{x_i'}\phi + u_{tu}\big), t = 1, \ldots, T$$

The key coefficient, β , captures the impact of Fall Armyworm (FAW) severity on the likelihood of household participation in charcoal production. The FAW variable is lagged by one year, reflecting that decisions to produce charcoal are influenced by FAW intensity in the previous agricultural season. Severe FAW-induced crop losses often push households to produce charcoal the following year to earn income for agricultural inputs. The model includes x_{it}, a vector of time-varying covariates such as household characteristics, agricultural production, and climatic factors, alongside $\underline{x_i'}$ (the Mundlak term), which accounts for potential correlation between covariates and unobserved individual heterogeneity. u_{it} is the idiosyncratic error term, and $\Phi(.)$ represents the standard normal cumulative distribution function used in the probit model.

Further, we employ a Tobit model with random effects to understand the effect of FAW on food security and the physical quantity of charcoal produced, due to the large presence of 0's in the data which creates a truncation/censoring effect we want to control for. To estimate the effect of FAW intensity on both food security outcomes and the quantity of charcoal produced, we employ the following Tobit model specification:

$$Q^*_{nt} = \beta' FAW_{nt-1} + \gamma X_{nt} + \omega_t + \zeta_{nt}$$

$$Q_{nt} = \begin{cases} Q^*_{nt} & \text{if } Q^*_{nt} > 0 \\ 0 & \text{other wise} \end{cases}$$

Here, subscript *n* refers to the household and *t* refers to time. Q_{nt} is the observed value of the outcome variable (either food security or charcoal production) for household *n* at time *t*, while Q^*_{nt} represents the latent (uncensored) value of the outcome. The parameter β' captures the effect of FAW intensity. The vector X_{nt} includes time-varying control variables such as household characteristics and climatic conditions. The term ω_t captures year-fixed effects to account for time-specific shocks common to all households.

The model assumes a random effects structure, where the composite error term is defined as:

$$\zeta_{nt} = \lambda_i + u_{nt}$$

In this expression, λ_i represents unobserved household-specific random effects, while u_{nt} is the idiosyncratic error term. The random effects λ_i are assumed to follow a normal distribution, independent of the explanatory variables, in line with standard assumptions (15).

For the rest of the regressions with use a two-way fixed effects estimation as specified below:

$$Y_{it} = \beta FAW_{it-1} + \gamma X_{it} + \mu_t + \alpha_i + \varepsilon_{it}$$

The dependent variable Y_{it} represents the outcome of interest, such as yield or cultivated land, all of which are continuous measures. X_{it} is a vector of time-varying explanatory variables. The term α_i captures the influence of unobserved, time-invariant factors specific to each household, while μ_t represents year fixed effects, accounting for shocks or trends affecting all households uniformly. Lastly, ε_{it} denotes the idiosyncratic error term.

S3.1.1 Instrumental variables

For the instrument to be valid, reporting errors across camps must be randomly distributed, uncorrelated with maize yields (exogenous), and correlated with household-reported infestations through spatial spillovers, controlling for household fixed effects. We test instrument strength using first-stage regression to ensure household infestation intensities correlate with neighboring farms' FAW levels.

If reporting errors are random, average camp infestation levels influence maize yields only through household infestation likelihood. Spatially correlated errors, possibly from varying FAW training, could violate the exclusion restriction. To validate this, we assess spatial correlation using Moran's I and exclude camps with no FAW. We also test misreporting correlations with farmer and camp characteristics, regressing household deviations in FAW reporting and characteristics (e.g., age, education, fertilizer use) against camp averages. A difference-in-differences approach evaluates infestation reporting deviations, using maize yield lags as the dependent variable.

The instrument is the camp-level average FAW intensity, excluding the observed household:

$$CA_{it} = [\sum_{i=1}^{n} FAW_{it}]/(n-1)$$

The instrument CA_{it} is exogenous, strongly correlated with household infestations through spatial spillovers, and uncorrelated with maize yields or unobserved variables, meeting the exclusion restriction and ensuring robustness.

S3.2 Methods used for the remotely sensed data analysis

The sudden arrival of the FAW to Africa in 2016, provides an exogenous and negative shock to maize productivity in the continent. This is the source of temporal variation we use as part of our identification strategy. The cross-sectional variation comes from the suitability that different regions have for the presence of the FAW. The estimation of the effect of the arrival of the FAW on deforestation using a standard two-way fixed effects approach, requires particularly strong assumptions in this setting. The parallel trends assumption is an assumption that this is not likely to hold, since the arrival of the FAW is a country-wide shock that could potentially affect a wide set of variables that are also correlated with land-use decisions and deforestation. This is particularly problematic given how much deforestation is correlated through space. Therefore, the estimation of the coefficient associated with the suitability to the presence of the FAW will be biased if we do not precisely control for all the factors that are spatially correlated with deforestation and change due to the arrival of the FAW.

Thus, our preferred method uses machine learning (ML) to generate a counterfactual of what deforestation would have been in the absence of the arrival of the FAW. This counterfactual is generated using a high-dimensional model that includes spatial lags for deforestation and all the other variables in the model. We include spatial lags starting from a 5 km distance threshold, up to 20 km. This allows us to take an agnostic approach to the selection of the spatial structure for the model, which is chosen based on the predictive power that the different spatial lags have. We train a ML model that will predict deforestation within each grid cell, as a function of past deforestation, the past spatial lags of deforestation, a set of control variables used in (2), and interactions between all of these variables. The set of control variables includes rainfall, temperature, potential yield for thirteen different crops, elevation, tree cover in 2001, population, and the travel time to the closest town with 50,000 inhabitants.

$$FL_{it} = \sum_{k=3}^{6} \beta_{1,k} FL_{it-k} \sum_{m=5}^{20} \sum_{k=3}^{9} \beta_{2,k} \rho_m FL_{it-k} + \sum_{k=1}^{9} \beta_{3,k} X_{it-k} + \sum_{m=5}^{20} \sum_{k=1}^{9} \beta_{4,k} \rho_m X_{it-k} \qquad (2)$$

Equation (2) is a linear function, where the forest loss (FL) in grid cell *i* in year *t*, will be modeled as a function of the previous deforestation in all the neighboring grid cells *m* kilometers around grid cell *i* ($\rho_m FL_{it-k}$), to capture the spatial effects associated with deforestation, as well as a set of control variables X_{it} is a vector of controls and $\rho_m X_{it}$ are the spatial lags of those control variables, which also includes interactions between all these variables. We then use (2) to train a model that produces an accurate out-of-sample prediction of forest loss in each grid cell, but we will only use data prior to the appearance of the FAW (i.e. 2017). We restrict the training period from 2001 to 2016, because the FAW was detected in West Africa in August 2016, and in Zambia in November 2016, so that forest loss in 2016 would not have been affected by the FAW in 2016.

Given the Lasso estimator (16):

$$\hat{\beta}(lasso) = arg\ arg\ min\ \|y - \sum_{j=1}^{p} X_j \beta_j\ \|^2 + \lambda \sum_{j=1}^{p} X_j \beta_j \qquad (3)$$

We use the training dataset and 1-step ahead cross-validation to find the *lambda* (from (3)) that minimizes the out-of-sample prediction error. The cross-validation algorithm works as follows. First, it takes all the data up to 2010, and selects the *lambda* that minimizes the prediction error when predicting forest loss for all the grid cells in 2011. It then moves up one year, and includes the data up to 2011, selects a *lambda* that minimizes the prediction error for 2012, and so on, until it reaches 2016. It then chooses the *lambda* that minimizes the out of sample prediction error for all the training period and generates a prediction for forest loss for each year and grid cell, from 2017 to 2019.

The most commonly used cross-validation method is k-fold cross-validation, which is not an appropriate cross-validation method in this case. This is because the spatial correlation of deforestation between grid cells implies that there could be contamination in the training sample, from the left out sample (the spatial lags from previous periods, which were included in the prediction, could contain some information of the observations that were left out for testing). This would imply that the out-of-sample performance of the models could be overestimated, and the *lambda* chosen using this approach would not minimize the out-of-sample prediction error (i.e., the model could be overfitted).

The one-step ahead cross-validation exercise allows us to find the optimal *lambda*, which is then used to select the covariates that lead to the best out-of-sample prediction. This is the model we

use to generate a prediction of the level of deforestation for each grid cell between 2017 and 2019. This predicted deforestation represents the counterfactual of what deforestation would have been in the absence of the FAW. Thus, the difference between the observed and predicted deforestation (i.e the prediction error) shows how deforestation within each grid cell changed as a consequence of the arrival of the FAW.

In order to estimate how the prediction error varies by the level of suitability to the FAW, we estimate the following fixed effects model:

$$FL_{it} - \widehat{FL}_{it} = \sum_{t=2010}^{2018} \beta_t Year_t \times Suitability_i + \rho_2 X_{it} + \rho_3 PredYield_{it} + year + \alpha_i + \varepsilon_{it} \quad (4)$$

The coefficients of interest βt, which estimate the effect that the arrival of the FAW had on deforestation, capturing the exposure of each grid cell to the arrival of FAW, by the suitability index.

Importantly, equation (4) includes a term for the predicted maize yield at the grid cell level ($PredYield_{it}$). This term represents the maize yield that would have been observed in each grid cell and each year in the absence of the FAW, given the local weather conditions throughout the year. By including it in the regression, we aim to control for the effect that weather has on deforestation, through its (predicted) effect on maize yields.

To generate this term, we use district level data from Zambia's crop forecast survey (CFS) (11). We trained a Lasso model using k-fold cross-validation and adaptive Lasso, only using observations prior to 2017, and randomly selected 80% of these observations as our training dataset, with the remaining 20% as our validation dataset. The k-fold cross-validation is based on 10 folds, with each fold representing a group of district and year level observations. The cross-validation method allows us to find the hyperparameter (lambda) that minimizes the out of sample prediction error (mean square error - MSE), since it iteratively trains the model on 9 of the 10 folds, and uses the left out fold to check the model's performance for a set of lambda values (each time, it uses 100 different values of lambda). The lambda that minimizes the out-of-sample mean square error (MSE) is chosen as the best possible lambda, and the choice of lambda will then determine what variables are selected for the final model. Adaptive Lasso implements a similar procedure, only that after lambda is chosen after the cross-validation process, an OLS model is estimated, after which the whole process is repeated, using the estimated coefficients as weights. A second lambda is then chosen, and this is the one that is used to do the final model selection.

There are three different outcome variables: maize yields based on the total area planted, maize yields based on the area that was actually harvested, and total maize production. The input variables are: monthly rainfall, temperature and normalized vegetation index, and the potential yield for thirteen crops, as well as interactions between all these variables, and a 1-year temporal lag of them. Given that the data we use to train the district level model is the same data we have at the grid cell level, we can then use the district level model, to generate a prediction of maize yields for each grid cell and year ($PredYield_{it}$), which is the term that we include in the regression equation (4).

S3.3 Spatial Bootstrapping procedure

The way in which we construct our dataset, by creating a grid that covers all of the country and using each grid cell as our unit of observation, allows us to have a very granular dataset. However, the high spatial correlation not only in deforestation but also in ecological characteristics and weather patterns, implies that these variables will be spatially correlated across the grid cells, and we need to know what the right spatial correlation structure is to be able to estimate the right

standard errors. Therefore, we implement a spatial bootstrapping procedure to estimate the standard errors. This procedure ensures that the grid cells used to estimate our models are far away from each other, so that they are unlikely to be spatially correlated with each other.

To do this, we create another grid which we overlay over Zambia. This grid has 0.25 by 0.25 degrees gridded cells, which we call the clustering cell for clarity, with a total of 1,123 clustering cells covering all of Zambia. Each gridded cell from our original dataset then falls inside one of the clustering cells (see Figure S6). We then randomly select a grid cell from each cluster cell, so that we know that the grid cells selected will be from clusters with centroids that are at least 0.5 degrees (~50 km) apart from the nearest cluster's centroid. We then estimate models (2) and (4) with the randomly drawn sample and store the estimated coefficients and standard errors. We repeat this process 200 times and then average the estimated effect and associated standard errors.

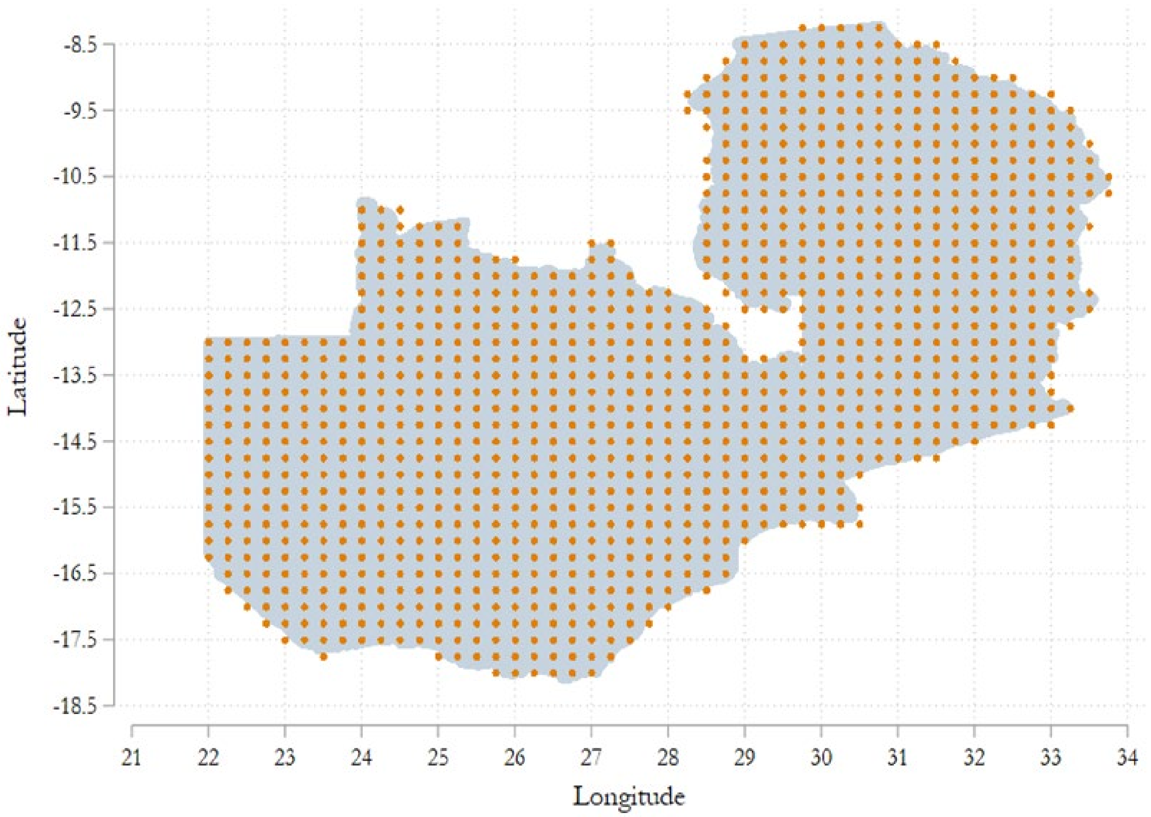

Figure S6. Centroids for the 25 km x 25 km clusters used for the spatial bootstrapping procedure.

SI References

1. J. Blekking, N. Gatti, K. Waldman, T. Evans, K. Baylis, The benefits and limitations of agricultural input cooperatives in Zambia. *World Development* **146**, 105616 (2021).
2. S. A. Spawn, C. C. Sullivan, T. J. Lark, H. K. Gibbs, Harmonized global maps of above and belowground biomass carbon density in the year 2010. *Sci Data* **7**, 112 (2020).
3. B. Vaitla, J. Coates, M. Daniel, "Comparing Household Food Consumption Indicators to Inform Acute Food Insecurity Phase Classification" (FHI 360/Food and Nutrition Technical Assistance III Project (FANTA), 2015).
4. M. C. Hansen, *et al.*, High-Resolution Global Maps of 21st-Century Forest Cover Change. *Science* **342**, 850–853 (2013).
5. I. M. McNicol, C. M. Ryan, E. T. A. Mitchard, Carbon losses from deforestation and widespread degradation offset by extensive growth in African woodlands. *Nat Commun* **9**, 3045 (2018).
6. R. Early, P. González-Moreno, S. T. Murphy, R. Day, Forecasting the global extent of invasion of the cereal pest Spodoptera frugiperda, the fall armyworm. *NB* **40**, 25–50 (2018).
7. C. Funk, *et al.*, The climate hazards infrared precipitation with stations—a new environmental record for monitoring extremes. *Sci Data* **2**, 150066 (2015).
8. NASA, MODIS Land Surface Temperature and Emissivity (MOD11). [Preprint] (2024). Available at: https://modis.gsfc.nasa.gov/data/dataprod/mod11.php [Accessed 1 December 2024].
9. NASA, MODIS Vegetation Index Products (NDVI and EVI). [Preprint] (2024). Available at: https://modis.gsfc.nasa.gov/data/dataprod/mod13.php [Accessed 1 December 2024].
10. Center For International Earth Science Information Network-CIESIN-Columbia University, Gridded Population of the World, Version 4 (GPWv4): Population Count, Revision 11. [Preprint] (2018). Available at: https://earthdata.nasa.gov/data/catalog/sedac-ciesin-sedac-gpwv4-popcount-r11-4.11 [Accessed 24 November 2024].
11. G. Fischer, *et al.*, Global Agro-ecological Zones (GAEZ v3.0) - Model Documentation. (2012). Available at: http://www.fao.org/soils-portal/soil-survey/soil-maps-and-databases/harmonized-world-soil-database-v12/en/ [Accessed 24 November 2024].
12. K. Mulungu, G. Tembo, H. Bett, H. Ngoma, Climate change and crop yields in Zambia: historical effects and future projections. *Environ Dev Sustain* **23**, 11859–11880 (2021).
13. G. Chamberlain, Multivariate regression models for panel data. *Journal of Econometrics* **18**, 5–46 (1982).
14. J. M. Wooldridge, Two-Way Fixed Effects, the Two-Way Mundlak Regression, and Difference-in-Differences Estimators. [Preprint] (2021). Available at: https://papers.ssrn.com/abstract=3906345 [Accessed 1 December 2024].
15. P. Kaya Samut, R. Cafrı, Analysis of the Efficiency Determinants of Health Systems in OECD Countries by DEA and Panel Tobit. Soc Indic Res 129, 113–132 (2016).
16. R. Tibshirani, Regression Shrinkage and Selection via the Lasso. *Journal of the Royal Statistical Society. Series B (Methodological)* **58**, 267–288 (1996).
